# Engaging natural regulatory myeloid cells to restrict T-cell hyperactivation-induced liver inflammation via extracellular vesicle-mediated purine metabolism regulation

**DOI:** 10.7150/thno.97427

**Published:** 2024-08-12

**Authors:** Fan Yang, Ruoting Men, Linling Lv, Leyu Zhou, Qiaoyu Deng, Xianglin Wang, Jingping Liu, Li Yang

**Affiliations:** 1 Department of Gastroenterology and Hepatology and Laboratory of Gastrointestinal Cancer and Liver Disease, West China Hospital of Sichuan University, Chengdu 610041, China.; 2 NHC Key Laboratory of Transplant Engineering and Immunology, Frontiers Science Center for Disease-related Molecular Network, West China Hospital of Sichuan University, Chengdu 610041, China.

**Keywords:** T cells, liver inflammation, purine metabolism, regulatory myeloid cells, extracellular vesicles

## Abstract

**Rationale:** Dysregulated T-cell immune response-mediated inflammation plays critical roles in the pathology of diverse liver diseases, but the underlying mechanism of liver immune homeostasis control and the specific therapies for limiting T-cell overactivation remain unclear.

**Methods:** The metabolic changes in concanavalin A (ConA) mice and autoimmune hepatitis (AIH) patients and their associations with liver injury were analyzed. The expression of purine catabolism nucleases (e.g., CD39 and CD73) on liver cells and immune cells was assessed. The effects of MCregs and their extracellular vesicles (EVs) on CD4^+^ T-cell overactivation and the underlying mechanism were also explored.

**Results:** Our findings revealed significant alterations in purine metabolism in ConA mice and AIH patients, which correlated with liver injury severity and therapeutic response. CD39 and CD73 were markedly upregulated on CD11b^+^Gr-1^+^ MCs under liver injury conditions. The naturally expanded CD39^+^CD73^+^Gr-1^high^CD11b^+^ MCreg subset during early liver injury effectively suppressed CD4^+^ T-cell hyperactivation and liver injury both in vitro and in vivo. Mechanistically, MCregs released CD73^high^ EVs, which converted extracellular AMP to immunosuppressive metabolites (e.g., adenosine and inosine), activating the cAMP pathway and inhibiting glycolysis and cytokine secretion in activated CD4^+^ T cells.

**Conclusions:** This study provides insights into the mechanism controlling immune homeostasis during the early liver injury phase and highlights that MCreg or MCreg-EV therapy may be a specific strategy for preventing diverse liver diseases induced by T-cell overactivation.

## Introduction

A dysregulated T-cell immune response and its associated uncontrolled inflammation play critical roles in the pathology of diverse liver diseases, such as autoimmune liver disease (AILD), nonalcoholic fatty liver disease (NASH), and liver cirrhosis 1. For example, substantial infiltration of CD4^+^ T cells and the release of excessive levels of proinflammatory cytokines, such as tumor necrosis factor-α (TNF-α) and interferon-γ (IFN-γ), along with consequent hepatocyte necrosis, have been extensively reported in the liver tissues of patients with autoimmune hepatitis (AIH) 2. T cells are crucial components of the adaptive immune system and can be further divided into various functional subsets, such as helper T cells and effector T cells. Upon exposure to proinflammatory stimuli (e.g., IFN-γ and IL-17), CD4^+^ T cells undergo overactivation and differentiate into effector T-cell populations (e.g., Th1 and Th17 cells), which can in turn cause direct (e.g., via cytotoxicity) or indirect injury (e.g., cytokine-mediated inflammation) to hepatocytes 3. Currently, the main clinical treatments for T-cell hyperactivation-related liver diseases (e.g., AIH) rely heavily on immunosuppressive drugs, such as glucocorticoids and azathioprine 4. However, these therapies exhibit limited efficacy and tolerability, and ~10%-20% of patients show an inadequate response to drugs, leading to disease progression 5, 6. Moreover, long-term use of immunosuppressive drugs (e.g., glucocorticoids) can suppress the overall immune system and increase the risk of secondary infection in these patients 7. Thus, novel therapies that can specifically inhibit T-cell hyperactivation while having minimal adverse effects on other immune cells are needed.

The maintenance of liver immune homeostasis relies on crosstalk between liver-resident immune cells and bone marrow (BM)-derived immune cells 8. For example, myeloid cells (MCs) undergo rapid differentiation and expansion in response to inflammatory stimuli in diverse liver diseases, such as autoimmune liver diseases and NAFLD 9, 10. BM-derived MCs are a group of heterogeneous cells containing several major subpopulations (e.g., granulocytes and mononuclear cells), and these cells can be rapidly mobilized and activated upon stimulation (e.g., pathogens, DAMPs), with elevated innate immune responses (e.g., phagocytosis, cytokine release) that aim to eliminate threats. Moreover, immature MCs can also differentiate into immunoregulatory myeloid cells (MCregs) under certain conditions, such as persistent infection or inflammation 11. Previous studies have revealed the existence of subsets of MCregs and their protective roles in several types of liver injury, including AIH 12. For example, CD11b- and Gr-1-positive MCs were shown to suppress CD4^+^ effector T-cell proliferation while inducing regulatory T-cell (Treg) expansion by secreting immunosuppressive factors (e.g., ARG1, iNOS, and IL-10) 12. Based on these findings, we speculated that certain MC subsets may also be expanded in response to T-cell hyperactivation during the early phase of liver injury, but its detailed features and underlying mechanisms are incompletely understood.

Abundant evidence has indicated the essential roles of metabolic pathways in modulating immune cell fate and functionality. For example, activated effector T cells undergo a metabolic shift from oxidative phosphorylation to glycolysis, which facilitates energy acquisition for cell proliferation and effector functions 13. Additionally, immunosuppressive MCs can restrict T-cell activation via metabolic regulation, such as by interfering with T-cell energy metabolism. For instance, immunosuppressive MCs were found to impede lactate efflux from CD4^+^ T cells and thus disrupt the metabolism and activation of CD4^+^ T cells 14. These reports suggest that metabolic state alteration is strongly associated with the phenotype and effector function of T cells and may serve as a potential target (metabolic checkpoint) for therapeutic intervention in T-cell-mediated liver inflammatory diseases. In recent years, mass spectrometry (MS)-based metabolomics analyses have been widely utilized to investigate metabolic perturbations and identify novel biomarkers of liver diseases 15, 16. Therefore, global profiling of metabolic alterations during the onset and progression of T-cell-mediated liver injury may provide insights into the intrinsic mechanisms that regulate liver immune homeostasis.

In the present study, to explore the immune-metabolic response that regulates liver immune homeostasis, we profiled metabolic changes in mouse models and patients with T-cell hyperactivation and revealed a strong association between alterations in purine metabolism and liver inflammation. We further identified a naturally expanded subset of MCregs (CD39^+^CD73^+^Gr-1^high^CD11b^+^) in the early phase of liver injury, and these cells suppressed CD4^+^ T-cell hyperactivation and related liver injury. The inhibitory effect of MCregs on CD4^+^ T-cell activation was at least partly due to the release of CD73^high^ extracellular vesicles (EVs), which convert extracellular AMP to immunosuppressive metabolites (e.g., adenosine and inosine) and activate the cAMP pathway in T cells. This study may improve our understanding of immune homeostasis control mechanisms during early liver injury and highlights that MCreg or MCreg-EV therapy may be a specific strategy for preventing effector T-cell-mediated liver diseases.

## Results

### Altered purine metabolism is a natural metabolic response during immune-mediated liver inflammation

In this study, a concanavalin A (ConA)-induced acute hepatitis model was used as a tool to explore metabolic changes in T-cell-mediated liver injury since ConA can induce antigen-nonspecific T-cell activation and severe liver inflammation and has been widely used to evaluate possible therapies for AIH 17. Additionally, the pathology of AIH is characterized by massive T-cell infiltration and activation, as well as diverse metabolic disruptions, such as increased lipolysis, protein hydrolysis, and glycolysis 18. We first evaluated the changes in liver injury in mice at a series of time points after ConA administration. The levels of liver injury indicators, such as plasma alanine aminotransferase (ALT) and aspartate aminotransferase (AST), peaked, and T-cell activation significantly increased in mice between 12 h and 24 h after ConA injection (Figure S1A-D). Hepatic histological examination revealed increased hepatocyte necrosis and apoptosis 24 h after ConA injection compared to 12 h, accompanied by increased histological injury scores (Figure S1E). Thus, 24 h post-ConA injection was selected as the timepoint for exploring the changes in early liver injury in subsequent experiments.

**Figure 1:**
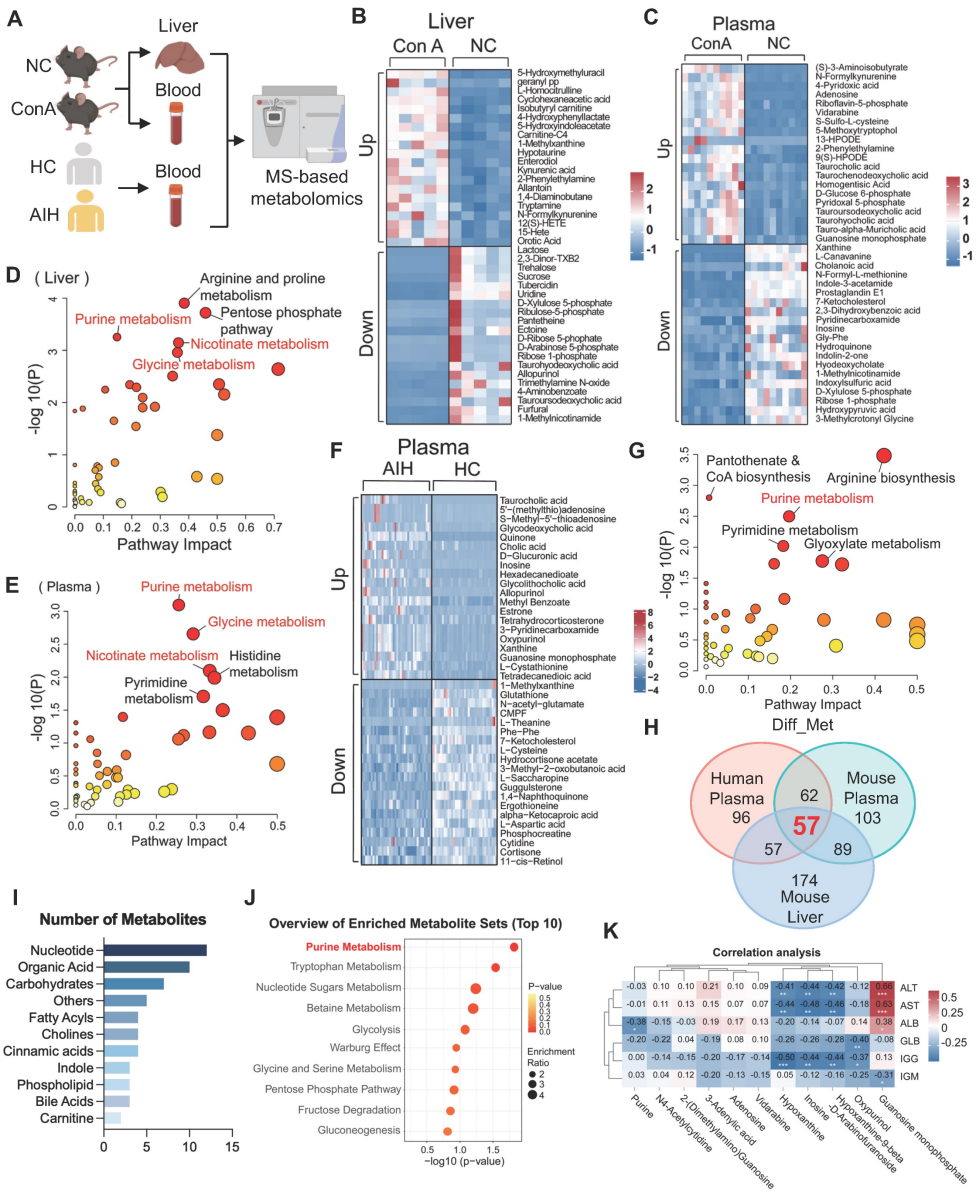
Purine metabolism is a potent metabolic checkpoint of T-cell hyperactivation-mediated liver injury. (A) Liver and plasma samples from ConA-induced mice and NC mice and plasma samples from AIH patients and healthy controls were collected for metabolomics. (B-C) The heatmap displays the top 20 up- and downregulated differentially abundant metabolites in mouse liver and plasma. (D-E) Pathway analyses based on the differentially abundant metabolites in mouse liver and plasma between the ConA and NC groups. (F) The heatmap displays the top 20 up- and downregulated differentially abundant metabolites in human plasma. (G) Pathway analyses based on the differentially abundant metabolites in human plasma between AIH patients and HCs. (H) The number of identical differentially abundant metabolites in liver and plasma samples from mice and humans. (I) The species of identical differentially abundant metabolites according to the three metabolomics analyses. (J) Pathway analyses based on the differentially abundant metabolites identified by the three metabolomics methods. (K) Pearson correlation coefficients (r) were calculated for the expression levels of purine nucleotides and clinical indicators. The numbers in the boxes are the r values. The depth of the color reflects the |r| value; red or blue indicates a positive or negative correlation, respectively.^ *^p < 0.05, ^**^p < 0.01, ^***^p < 0.001.

As shown in Figure 1A, liver and plasma samples from mice (ConA and normal control [NC] groups) and plasma samples from humans (AIH and healthy control [HC] groups) were collected and then subjected to LC-MS-based metabolomics analysis. A principal component analysis (PCA) scatter plot showed a distinct separation between the ConA and NC groups and between the AIH and HC groups (Figure S2A-C). Volcano plot and heatmap showing the significantly changed metabolites (|log2FC| > 0, and p value < 0.05) in liver or plasma samples between ConA and NC mice (Figure S2D-E and S2G-H). The differentially abundant metabolites are listed in Tables S1 and S2. Heatmaps illustrating the top 20 upregulated and downregulated metabolites, including amino acids, organic acids, nucleotides and fatty acyls, between the ConA and NC groups are shown in Figure 1B-C. Previous studies have revealed substantial perturbations in amino acid, carbohydrate, and nucleotide purine metabolism across various AIH genotypes 19. Kyoto Encyclopedia of Genes and Genomes (KEGG) pathway enrichment analyses revealed that the altered metabolites in liver or plasma samples of the ConA group were enriched mainly in pathways related to purine metabolism, glycine metabolism, and nicotinate metabolism (Figure 1D-E).

Similarly, a volcano map and heatmap showed many differentially abundant metabolites in the plasma samples of AIH patients compared to those in the plasma samples of HCs (Figure 1F, S2F and S2I), and these differentially abundant metabolites are listed in Table S3. Furthermore, these altered metabolites were also predominantly enriched in pathways related to purine metabolism, arginine metabolism, pyrimidine metabolism, etc. (Figure 1G). To identify the shared metabolic changes between ConA mice and AIH patients, we analyzed the three metabolomics datasets and identified 57 differentially abundant metabolites (Figure 1H). Notably, these commonly altered metabolites, such as nucleotides and organic acids, were mainly enriched in pathways related to purine metabolism, tryptophan metabolism, etc. (Figure 1I-J). The expression patterns of these differentially expressed purine nucleotides between the AIH and HC groups are displayed in Figure S3. The possible association between purine metabolism and liver injury in AIH patients was further evaluated by correlating the differential nucleotides involved in purine metabolism with liver function indicators (ALT, AST, and ALB) and immunological indices (globulin [GLB], immunoglobulin G [IGG], and immunoglobulin M [IGM]). There was a significant negative correlation between the levels of purine metabolites (e.g., inosine, hypoxanthine and oxypurinol) and several clinical indicators, such as ALT, AST and IGG (Figure 1K). Together, these results suggest that altered purine metabolism may be a metabolic regulator involved in immune-mediated liver injury.

### Upregulation of the purinergic signaling axis in MCs under T-cell hyperactivation conditions *in vivo*

**Figure 2:**
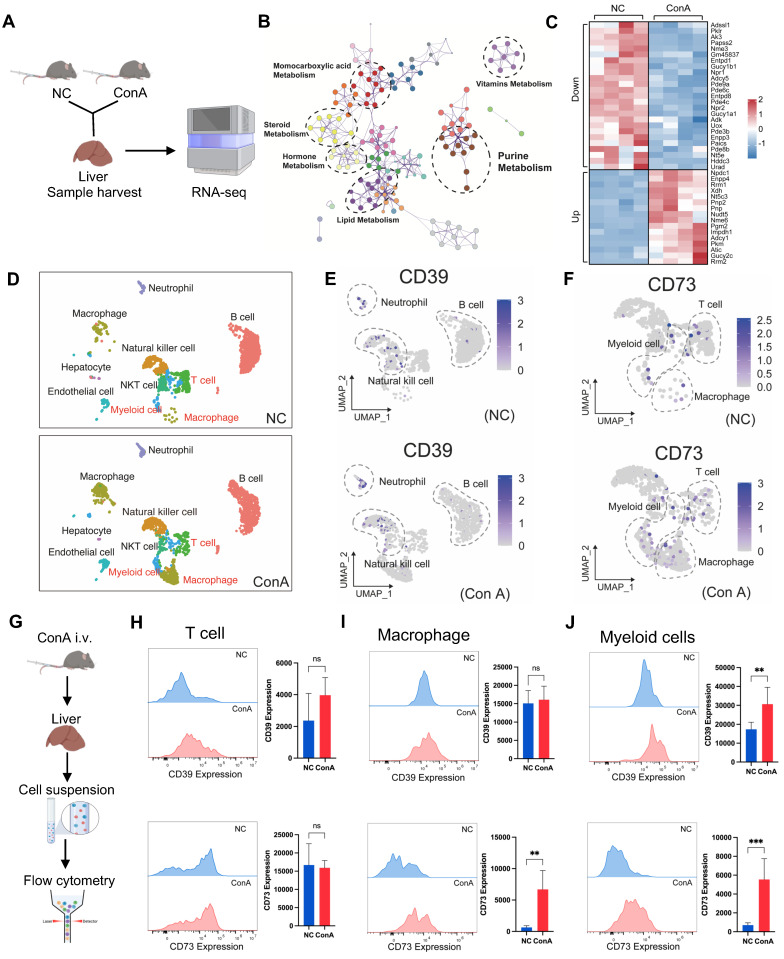
Upregulation of the CD39-CD73 axis in MCs under T-cell activation-mediated liver injury conditions. (A) Livers from ConA and NC mice were collected for bulk RNA sequencing. (B) Pathway analyses based on the DEGs between the NC and ConA groups. (C) Total differentially expressed genes involved in purine metabolism between the ConA group and the NC group (n = 4 per group). (D) Differences in specific cell clusters between the NC and ConA groups (n = 3 per group). (E-F) The differential expression of CD39 and CD73 in distinct cell clusters between the NC and ConA groups. (G) Validation of CD39 and CD73 expression on immune cells by flow cytometry. (H-J) CD39 and CD73 expression in CD3-positive T cells, CD11b- and F4/80-positive macrophages, and CD11b- and Gr-1-positive MCs between the ConA and NC groups (n = 5 per group).

To explore the pathway underlying the altered purine metabolism, we conducted bulk RNA-seq analysis of mouse liver tissues (Figure 2A). A PCA scatter plot showed clear separation between the ConA and NC groups (Figure S4A). A number of DEGs (1876 upregulated DEGs and 2011 downregulated DEGs) were identified (Figure S4B and Table S4); these DEGs were enriched mainly in pathways related to purine metabolism, lipid metabolism, etc. (Figure 2B), and multiple DEGs of liver tissues in the ConA group compared to those in the NC group were key metabolic genes involved in regulating purine metabolism (with 16 upregulated and 25 downregulated genes), such as Entpd1 (encoding the CD39 protein) and Nt5e (encoding the CD73 protein) (Figure 2C).

CD39 and CD73 are cell surface nucleases that regulate purine metabolism. CD39 can convert extracellular ATP to AMP, followed by the conversion of AMP to adenosine by CD73, and this process can promote a shift from a proinflammatory state to an anti-inflammatory state in immune cells 20. Next, we sought to determine the specific cell types within liver tissues involved in regulating purine metabolism. We first detected altered transcript levels of CD39 and CD73 in mouse liver parenchymal (hepatocytes) and nonparenchymal cells (Figure S5A). The results showed that CD39 and CD73 expression was downregulated in mouse hepatocytes, consistent with the bulk sequencing data (Figure S5B). However, the expression in nonparenchymal cells contrasted with the sequencing results, suggesting that some of these cells may be involved in regulating purine metabolic homeostasis (Figure S5C).

Previous studies have reported that CD39 and CD73 might be primarily expressed on immune cells, such as monocytes and macrophages 20. Thus, we analyzed the public single-cell RNA-seq data of liver tissues from ConA and NC mice 21, minimizing the influence of hepatocytes on transcriptional outcomes. The analysis revealed a significant increase in the proportions of T cells, macrophages, and MCs in the ConA group compared to those in the NC group, and the CD39 and CD73 genes were predominantly expressed in these three cell types (Figure 2D-F). Further flow cytometry analysis (FCA) was used to detect the expression of CD39 and CD73 in T cells, macrophages and MCs (Figure 2G). Compared with T cells (CD3-positive) and macrophages (CD11b- and F4/80-positive), CD11b- and Gr-1-positive MCs showed markedly elevated CD39 and CD73 expression (Figure 2H-J). Although CD73 expression was upregulated on macrophages, previous studies have revealed a predominant role of proinflammatory macrophage polarization in the early stage of ConA-induced liver injury 22. Similarly, our results also showed that the increase in hepatic macrophages in the ConA group was predominantly F4/80- and CD86/iNOS-positive M1-polarized, with no significant changes in the proportions of F4/80- and CD206-positive M2-polarized macrophages (Figure S6A-D), suggesting that CD73^+^ M1-like macrophages are unlikely to play an immunosuppressive role. Together, these results suggest that the CD39-CD73 axis, particularly in MCs, plays a regulatory role in purine metabolism during the onset of T-cell-induced liver injury.

### Natural expansion of a regulatory MC subset in response to liver inflammation

**Figure 3:**
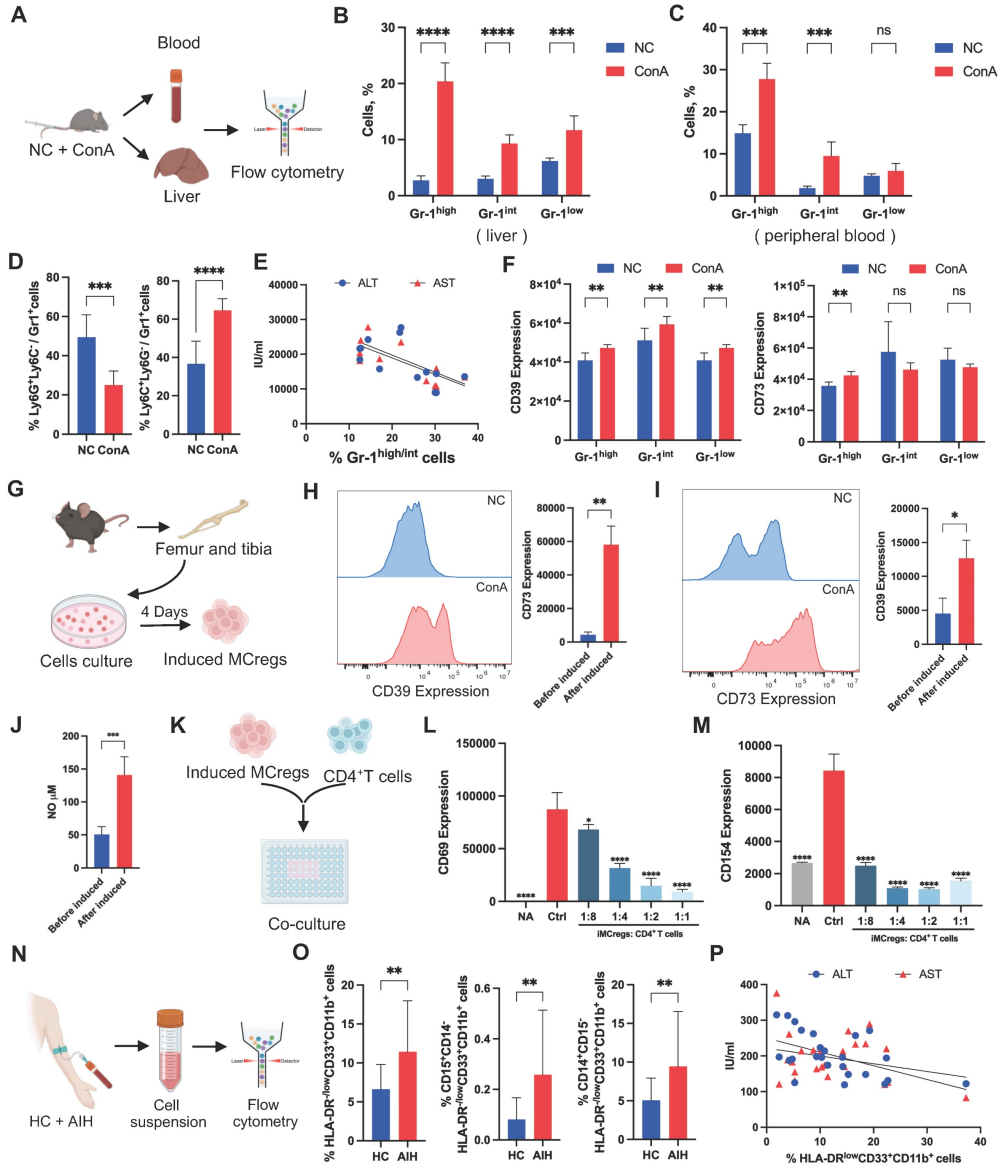
Natural expansion of a regulatory MC subset in response to liver inflammation. (A) Blood and livers from ConA-induced and NC mice were collected for the detection of MCregs. (B-C) The distribution of CD11b^+^Gr-1^high^, Gr-1^int^, and Gr-1^low^ cells in the liver and blood (n = 5-7). (D) Proportion of Ly6C^+^ mononuclear lineage and Ly6G^+^ granular lineage cells within the CD11b^+^Gr-1^high/int^ cell population in the liver (n = 5-7). (E) Correlation analysis revealed a negative correlation between the percentage of CD11b^+^Gr-1^high/int^ cells and aminotransferase levels (ALT and AST). (F) Expression levels of CD39 and CD73 in CD11b^+^Gr-1^high^, Gr-1^int^, and Gr-1^low^ cells. (G) The process of MCreg isolation and induction (n = 5-7). (H-I) Changes in CD39 and CD73 expression on MCs before and after induction. (J) The release of NO in MCs before and after induction. (K) The induced MCregs were cocultured with CD4^+^ T cells at 1:8, 1:4, 1:2, and 1:1 ratios. (L-M) Changes in the expression of CD69 and CD154, which are indicative of T-cell activation, on CD4^+^ T cells after coculture with induced MCregs at various ratios. (N) Blood from HCs and AIH patients was collected for the evaluation of human MCregs. (O) Percentage of HLA-DR^-/low^CD33^+^CD11b^+^ cells in peripheral blood, along with the distribution of the CD14^+^ mononuclear lineage and CD15^+^ granulocytic lineage within these cells. (P) Correlation analysis between the percentage of HLA-DR^-/low^CD33^+^CD11b^+^ cells and clinical indicators (ALT and AST).

Many previous studies have shown the protective role of certain MC subsets in immune-related disease progression 12, but the underlying mechanism is not fully understood. CD11b and Gr-1 are common cell surface markers of MCs in mice. Glutathione reductase (Gr-1) is a family of antigens that includes Ly6G and Ly6C. Ly6G is primarily expressed on granulocytes, while Ly6C is found on monocytes and macrophage subsets 23, which led to the identification of two distinct MC subpopulations, granulocytic and monocytic MC lineages. Based on the cellular Gr-1 expression levels, we classified CD11b^+^Gr-1^+^ cells from mouse liver and peripheral blood into Gr-1^high^, Gr-1^int^, and Gr-1^low^ groups (Figure 3A and S7A). The results showed a notable upregulation of Gr-1^high^ and Gr-1^int^ cell populations in liver tissues or peripheral blood from the ConA group compared to the NC group (Figure 3B-C), and the predominant cell subset was mononuclear lineage MCs (Figure 3D and S7B).

To explore the possible role of Gr-1^high/int^ MC subsets in T-cell-mediated liver injury, a correlation analysis between the Gr-1^high/int^ MC populations and liver function indicators (ALT and AST) in ConA mice was performed. Interestingly, we found a significant negative correlation between the percentage of Gr-1^high/int^ MC subsets and ALT/AST levels, suggesting that these MCs may play protective roles in T-cell-mediated liver injury (Figure 3E). Further FCA revealed that the Gr-1^high^ MC subsets had more pronounced changes in CD39 and CD73 expression than did the Gr-1^int^ and Gr-1^low^ MC subsets (Figure 3F). Thus, the expansion of CD39^+^CD73^+^Gr-1^high^CD11b^+^ MC subsets (MCregs) might be a natural response of the immune system to restrict T-cell-mediated liver inflammation *in vivo*.

To assess the functionality of this MC subset, MCs isolated from the bone marrow of normal mice were induced with granulocyte colony-stimulating factor (G-CSF) and interleukin 6 (IL-6) *in vitro* as previously reported (Figure 3G) 24. The induced MCregs (iMCregs), such as Gr-1, CD39 and CD73, partially replicated the phenotype of CD39^+^CD73^+^Gr-1^high^CD11b^+^ MCs observed *in vivo*. The expression of Gr-1 on the iMCregs was upregulated (Figure S8A), with notable upregulation of CD39 and CD73 expression on the iMCregs (Figure 3H-I). The expression of key enzymes involved in MC-mediated immunosuppression, such as inducible nitric oxide synthase (iNOS), NADPH oxidase 2 (NOX2), and arginase (ARG1) 25, was significantly elevated in MCregs (Figure S8B). Moreover, iMCregs exhibited a notable increase in nitric oxide (NO) release (Figure 3J), which has also been involved in MC-mediated T-cell proliferation inhibition or T-cell apoptosis induction 25. To further evaluate the immunomodulatory capacity of iMCregs, we cocultured them with CD4^+^ T cells at ratios of 1:8, 1:4, 1:2, and 1:1 (Figure 3K). Compared to coculture with CD4^+^ T cells alone, coculture with iMCregs inhibited CD4^+^ T-cell activation, as evidenced by reduced levels of CD69 and CD154, in a dose-dependent manner (Figure 3L-M). The most significant inhibitory effect was observed at a 1:1 ratio of iMCregs to CD4^+^ T cells.

Unlike those in mice, the immunosuppressive MCregs in humans were identified as HLA-DR^-^/^low^CD33^+^CD11b^+^ cells, which were further classified into monocytic and polymorphonuclear cells based on CD14 and CD15 expression 26. Similarly, a substantial increase in the number of MCregs with increased proportions of mononuclear (CD14-positive) and granulocytic (CD15-positive) lineages was observed in the peripheral blood of AIH patients (Figure 3N-O and S9). There was a significant negative correlation between the proportions of MCregs and liver function indicators (ALT and AST) in AIH patients (Figure 3P), suggesting that these human MCregs may also play immunoregulatory roles during liver injury.

### MCregs play an immunomodulatory role in the liver by regulating purine catabolism

**Figure 4:**
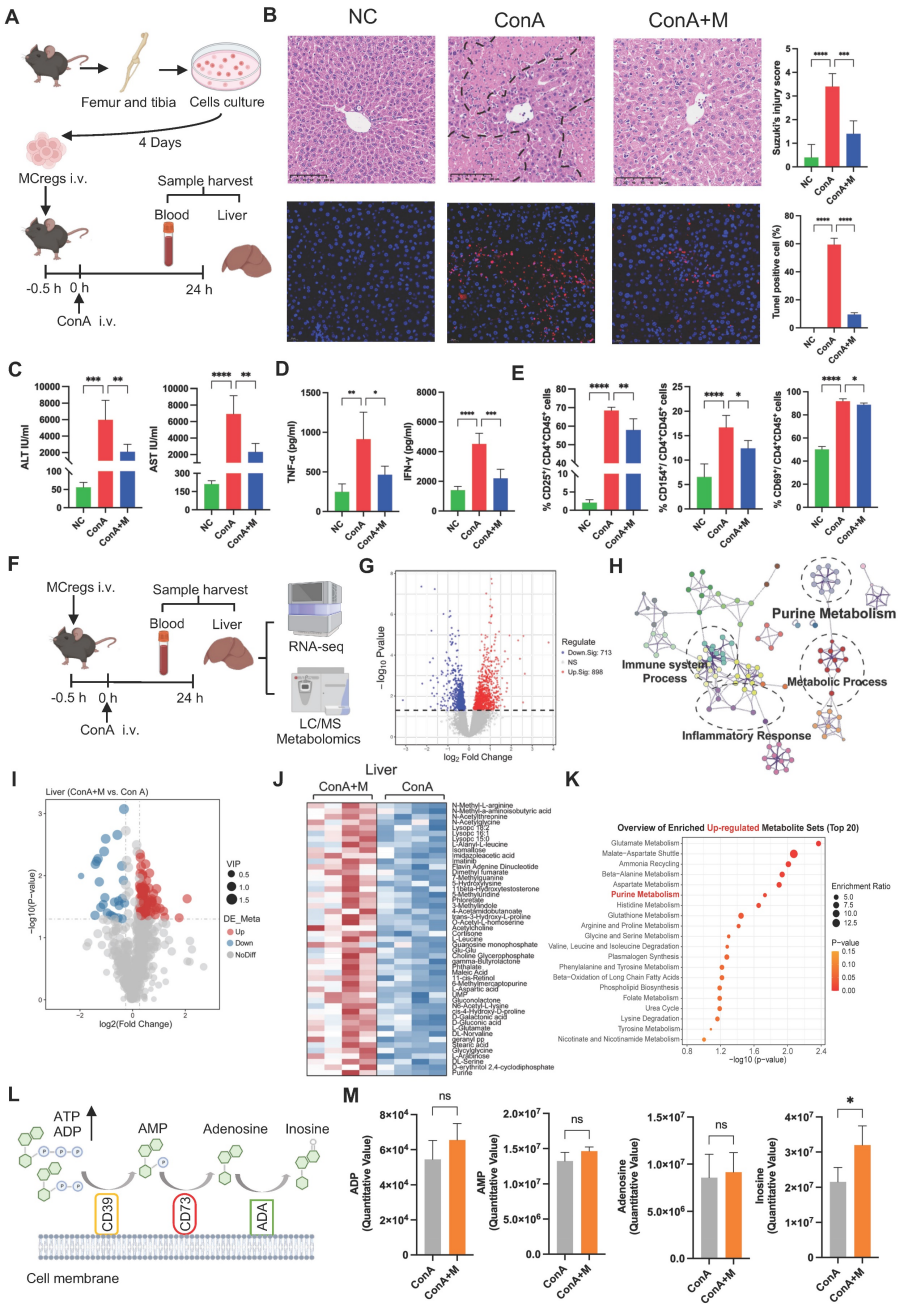
MCregs play an immunomodulatory role in the liver by regulating purine catabolism. (A) After the adoptive transfer of MCregs into ConA-induced mice, the liver and blood were collected for subsequent analyses. (B) Hepatic tissues from the NC, ConA, and MCreg-treated groups (ConA+M) were subjected to H&E and TUNEL staining. H&E staining was used to assess the Suzuki injury score, while apoptotic cell ratios were determined via TUNEL staining (scale bar = 20 µm, n = 5). (C) Detection of the serum levels of ALT and AST in the mice (n = 5-6). (D) Detection of the serum levels of TNF-α and IFN-γ in the mice (n = 5-6). (E) The percentages of CD25-, CD69- and CD154-positive activated hepatic CD4^+^ T cells among the three groups (n = 5-6). (F) Bulk-RNA-seq and metabolomic analyses of blood and liver samples from the ConA and ConA+M groups (n = 4). (G) Volcano plot showing the DEGs between the ConA+M and ConA groups. (H) Pathway analyses based on the DEGs between the ConA+M and ConA groups. (I) Volcano plot showing the differentially metabolites between the ConA+M and ConA groups. (J) The top 50 upregulated differentially abundant metabolites in the liver between the ConA+M and ConA groups. (K) Pathway analyses based on the upregulated differentially abundant metabolites between the two groups. (L) Illustration of the purine metabolism process, involving the catabolism of ATP and ADP to AMP by CD39, which is further metabolized to adenosine (ADO) by CD73 and inosine (INO) by adenosine deaminase (ADA). (M) Comparison of the levels of ADP, AMP, ADO, and INO in the liver between the ConA and ConA+M groups.

To assess the therapeutic effects of MCregs, we adoptively transferred iMCregs into ConA-induced mice via tail vein injection (Figure 4A). A number of CM-Dil-labeled iMCregs were observed in mouse liver tissues 24 h after iv injection (Figure S10A), suggesting the robust ability of these cells to mitigate liver injury. Compared with ConA treatment alone, MCreg treatment reduced liver apoptosis/necrosis (Figure 4B) and the levels of serum ALT/AST in ConA-induced model (Figure 4C). Additionally, MCreg treatment decreased the serum levels of TNF-α and IFN-γ in ConA-induced mice (Figure 4D). Moreover, MCreg treatment inhibited CD4^+^ T-cell activation, as evidenced by lower numbers of activated T cells (as indicated by CD25, CD69, and CD154) in the livers of MCreg-treated mice than in those of ConA-treated mice (Figure 4E and S10B).

To explore the possible mechanisms underlying the immunomodulatory effects of MCregs *in vivo*, we conducted transcriptomic and metabolomic analyses on the livers and plasma of mice induced with ConA and treated with MCregs (Figure 4F). PCA demonstrated clear differences between the ConA and MCreg treatment groups (Figure S11A). We identified a significant number of DEGs (898 upregulated and 713 downregulated DEGs) affected by MCreg treatment (Figure 4G and Table S5). These DEGs were predominantly enriched in pathways associated with purine metabolism and inflammatory responses (Figure 4H). Further enrichment analysis of the downregulated DEGs in the MCreg-treated group revealed significant enrichment of inflammation-related pathways, including the TNF-α and NF-kB signaling pathways, as well as the IL-2 signaling pathway associated with T-cell proliferation (Figure S11B). However, there were no significant alterations in the TGF-β signaling pathway, Jak-STAT signaling pathway, or IL-17 signaling pathway (Figure S11C-E).

Next, the metabolic changes in liver and plasma samples from MCreg-treated and ConA-treated mice were analyzed via metabolomics. PCA plots clearly revealed differences between the two groups (Figure S12A-B), and volcano plots revealed differentially abundant metabolites (Figure 4I and S12C), suggesting that the immunomodulatory effects of MCregs occur through metabolic pathways. The differentially abundant metabolites are listed in Table S6 and S7. The top 50 upregulated metabolites in the liver tissues of MCreg-treated ConA mice were enriched in multiple pathways, such as purine metabolism and glutamate metabolism pathways (Figure 4J-K). In purine catabolism processes, ATP and ADP are converted to AMP via CD39, and AMP is catabolized to adenosine via CD73 and further to inosine via adenosine deaminase (ADA) (Figure 4L). In liver tissues, the MCregs had higher levels of inosine and slightly elevated levels of ADP, AMP, and adenosine than ConA group (Figure 4M). Compared with those of the ConA group, the plasma samples of the MCreg group also exhibited changes in the levels of purine catabolism metabolites, such as increased levels of inosine and decreased levels of adenosine (Figure S12D-E). The reduced adenosine levels in the MCreg-treated group might have been due to elevated blood ADA enzyme activity (Figure S12F). These results suggest that MCreg treatment can alleviate T-cell-mediated liver injury and that this effect likely occurs via the regulation of purine catabolism.

**Figure 5:**
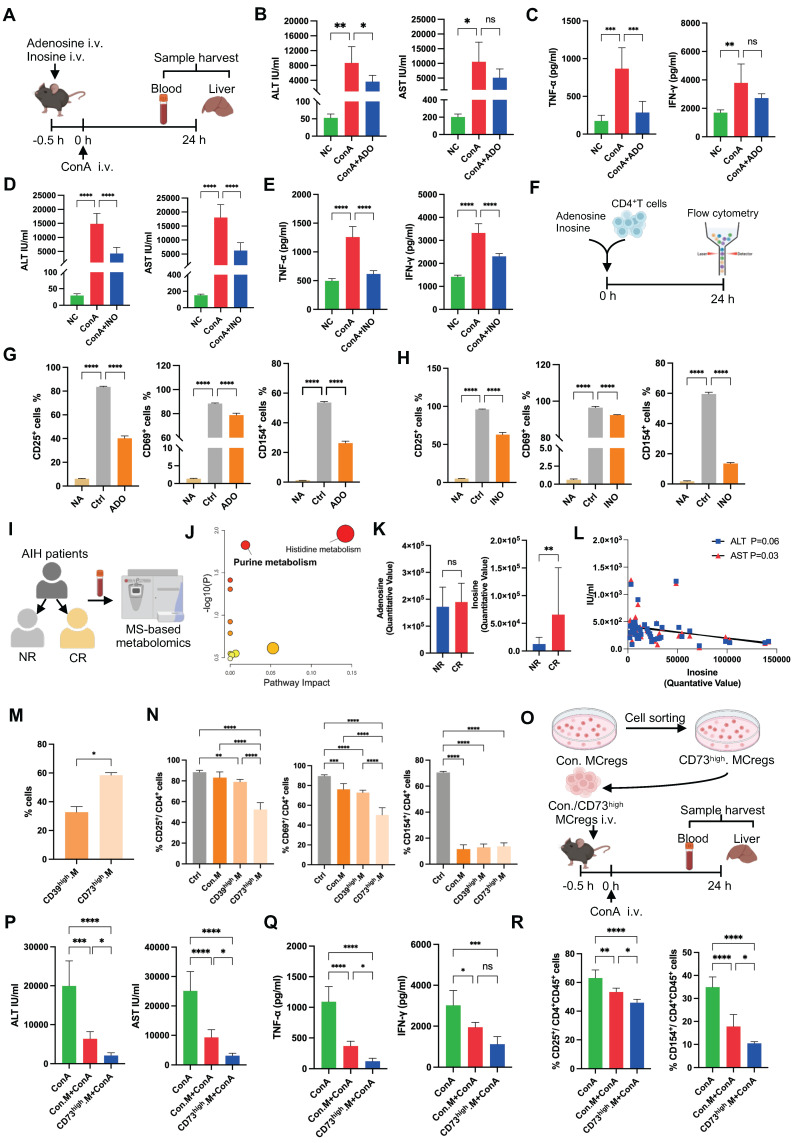
Immunomodulatory effects of MCregs via the CD73-adenosine/inosine axis. (A) Adenosine (ADO) and inosine (INO) were injected into ConA-induced mice via the tail vein, and the livers and blood were collected for subsequent analyses. (B-C) Serum levels of ALT/AST and TNF-α/IFN-γ in the different groups (n = 4-6). (D-E) Serum levels of ALT/AST and TNF-α/IFN-γ in the different groups (n = 4-6). (F) Flow chart of ADO and INO added to the culture of CD4^+^ T cells. (G) The percentages of CD25^+^, CD69^+^ and CD154^+^ activated CD4^+^ T cells among the untreated group (NA), control group (Ctrl) and ADO group. (H) The percentages of CD25^+^, CD69^+^ and CD154^+^ cells among activated CD4^+^ T cells in the NA group, Ctrl group and INO group. (I) AIH patients were categorized into nonresponsive (NR) and complete response (CR) groups, followed by plasma metabolomics analysis. (J) Pathway analyses based on the differentially abundant metabolites between the two groups. (K) The levels of ADO and INO in patients in the two groups. (L) Correlation analysis between INO levels and aminotransferase levels (ALT and AST). (M) The percentages of CD39 high-expressing (CD39^high^) and CD73 high-expressing (CD73^high^) MCregs before flow-sorting. (N) The percentages of CD25^+^, CD69^+^ and CD154^+^ cells among activated CD4^+^ T cells cultured with conventional (Con), CD39^high^ and CD73^high^ MCregs. (O) Flow-sorted CD73^high^ MCregs were intravenously injected 0.5 h before ConA administration. Samples were harvested 24 h post-ConA administration. (P-Q) Serum levels of ALT/AST and TNF-α/IFN-γ in the ConA group, Con.MCreg-treated group (Con.M+ConA) and CD73^high^M+ConA group (n = 5-6). (R) The percentages of CD25^+^ and CD154^+^ activated CD4^+^ T cells in these three groups (n = 5-6).

To confirm these findings, ConA-induced mice were treated with adenosine (ADO) or inosine (INO) via tail vein injection (Figure 5A), and ADO or INO treatments decreased the levels of serum transaminases (ALT and AST) and inflammatory cytokines (IFN-γ and TNF-α) in ConA-induced mice (Figure 5B-E). Furthermore, they inhibited liver CD4^+^ T-cell activation in ConA-treated mice (Figure S13A).* In vitro*, ADO or INO treatment also inhibited CD4^+^ T-cell activation without affecting T-cell viability (Figure 5F-H and S13B). The plasma metabolic profiles of AIH patients in the complete response (CR) and nonresponse (NR) groups were assayed based on their response to drug therapy (Figure 5I). A PCA scatter plot showed a trend of discrimination between the two groups, and a volcano plot showed the differentially abundant metabolites (Figure S14). The differentially abundant metabolites are listed in Table S8. The significantly altered metabolites were enriched in pathways related to purine metabolism and histidine metabolism (Figure 5J). Patients in the CR group had elevated plasma levels of ADO and INO compared to those in the NR group (Figure 5K), and there was a significant negative association between plasma inosine levels and liver function indicators (Figure 5L), suggesting the beneficial role of purine catabolism in patients' response to treatment.

To further verify that MCregs play an immunomodulatory role by regulating purine metabolism, we sorted CD39 high-expressing (CD39^high^) and CD73 high-expressing (CD73^high^) MCregs using flow cytometry. The sorting gates were shown in Figure S15A. The results showed that the percentage of CD73^high^ MCregs was greater than that of CD39^high^ MCregs (Figure 5M). Compared with conventional MCregs, both CD39^high^ and CD73^high^ MCregs significantly inhibited CD4^+^ T-cell activation, and CD73^high^ MCregs exhibited stronger immunosuppressive effects than did CD39^high^ MCregs (Figure 5N and S15B). Thus, CD73^high^ MCregs were sorted from total MCregs and then injected into ConA-induced mice (Figure 5O). Treatment with CD73^high^ MCregs more effectively reduced ALT and AST levels, as well as TNF-α and IFN-γ levels, in ConA-induced mice (Figure 5P-Q). Furthermore, CD73^high^ MCreg therapy effectively inhibited liver CD4^+^ T-cell activation in ConA-induced mice (Figure 5R and S15C). Together, these results indicate the immunomodulatory role of MCregs, and this effect may be due to the regulation of purine catabolism (e.g., CD73).

### MCregs restrict T-cell hyperactivation via EV-mediated metabolic regulation

**Figure 6:**
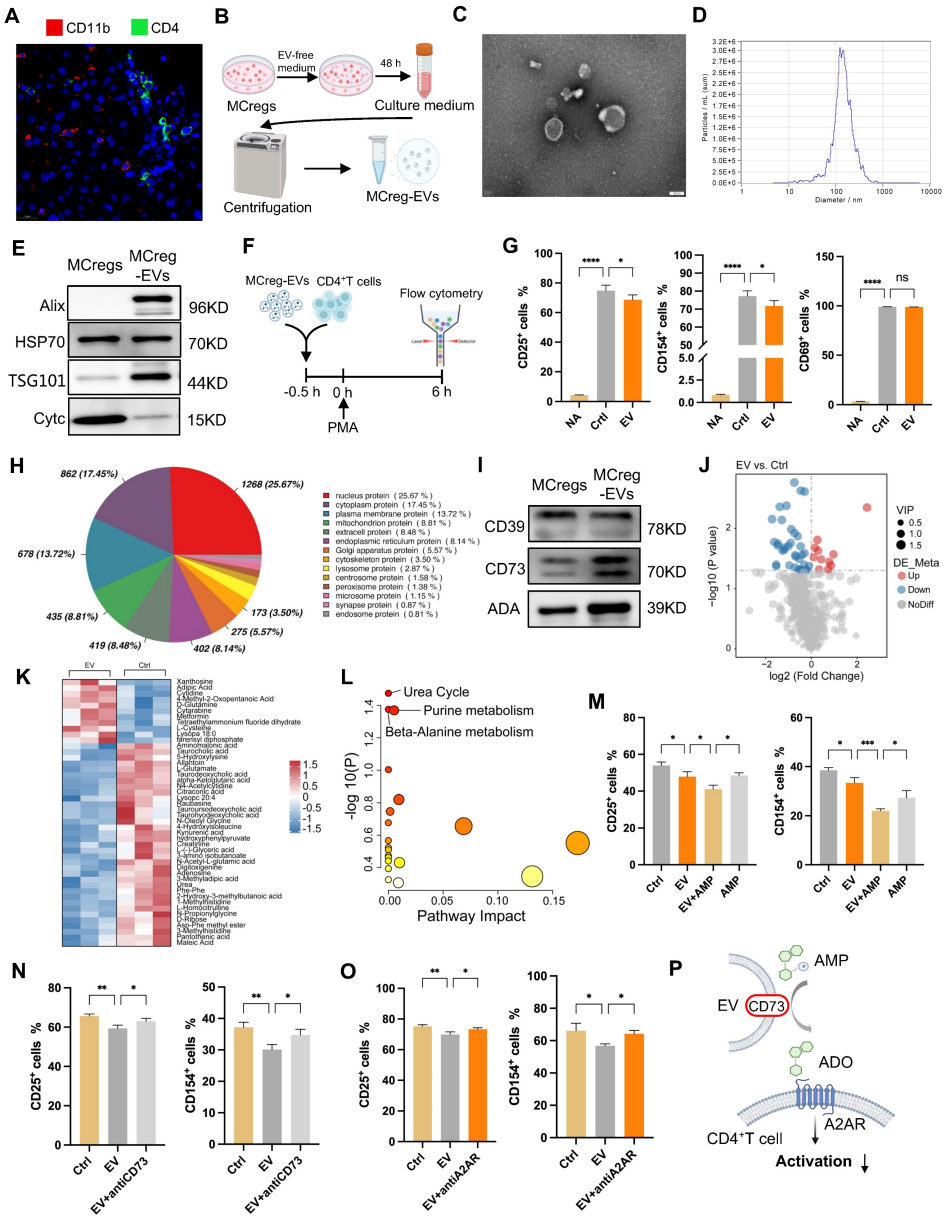
MCregs restrict T-cell activation through EV-mediated metabolic regulation. (A) Immunofluorescence showing MCregs and CD4^+^ T cells in ConA-induced livers. Red represents CD11b, which indicates MCregs, and green represents CD4, which indicates CD4^+^ T cells (scale bar = 20 µm, n = 5). (B) Induced MCregs were cultured in EV-free medium, and cell supernatants were collected after 48 h for EV isolation through ultracentrifugation. (C and D) TEM (scale bar = 100 nm) and NTA images of MCreg-derived EVs. (E) Evaluation of EV marker protein (Alix, HSP70, TSG01, and Cytc) expression levels. (F) CD4^+^ T cells pretreated with MCreg-EVs for 0.5 h were activated with PMA, and T-cell activation was measured by flow cytometry 6 h later. (G) Percentages of CD25^+^, CD69^+^, and CD154^+^ activated CD4^+^ T cells among the untreated group (NA), control group (Ctrl), and MCreg-EV-treated group (EV). (H) Types of proteins encoded by MCreg-EVs. (I) Protein levels of CD39, CD73, and ADA on MCregs and MCreg-EVs. (J) Volcano map showing the differentially abundant metabolites between the EV and Ctrl groups. (K) Heatmap illustrating the differentially abundant metabolites between the EV and Ctrl groups. (L) Pathway analyses based on the differentially abundant metabolites between the two groups. (M) Percentage of CD25^+^ and CD154^+^ cells in the Ctrl group, EV-only group, EV plus AMP group (EV+AMP), and AMP-only group. (N) Percentage of CD25^+^ and CD154^+^ cells in the Ctrl group, EV-only group, and EV plus CD73 inhibitor group (EV+anti-CD73). (O) Percentage of CD25^+^ and CD154^+^ cells in the Ctrl group, EV-only group, and EV plus A2AR inhibitor group (EV+antiA2AR). (P) AMP is degraded to ADO through CD73^+^ EVs, activating the A2AR receptor and thereby inhibiting CD4^+^ T-cell activation.

Immune cells can impact other cell functions through direct (cell-to-cell attachment) or paracrine (release of cytokines or EVs) mechanisms 12, 27. Thus, we first assessed the spatial relationship between MCregs and CD4^+^ T cells in the liver tissues of ConA mice using immunofluorescence. The results showed that many MCregs (red) did not colocalize with CD4^+^ T cells (green) in injured liver tissues (Figure 6A), suggesting that MCregs may exert their regulatory effect on T cells via a paracrine mechanism.

Notably, EVs are pivotal mediators of *in vivo* intercellular communication in diverse diseases, and we also observed abundant numbers of EVs in the liver tissues of ConA mice. Liver tissue-derived EVs were measured by nanoparticle tracking analysis (NTA), transmission electron microscopy (TEM) and western blotting (Figure S16). MCreg-derived EVs were isolated, and their structures and sizes were measured by TEM and NTA; positive protein markers (Alix, HSP70 and TSG101) and negative protein markers (Cytc) were detected by western blotting (Figure 6B-E). MCreg-EV treatment reduced the percentage of CD25- and CD154-positive activated CD4^+^ T cells (Figure 6F-G), suggesting that MCregs may exert immunosuppressive effects via EV release. However, MCreg-EV treatment did not significantly affect the proportion of regulatory T cells among activated CD4^+^ T cells (Figure S17A).

To further clarify their immunomodulatory role, we performed proteomic analysis of MCreg-EVs. The results revealed that the highly abundant EV proteins related to cell structure and function—specifically, nucleus, cytoplasm, and membrane proteins—contained CD39, CD73, and ADA, which are related to purine metabolism (Figure 6H). KEGG analysis revealed that proteins in MCreg-EVs were enriched in metabolism-related pathways, such as lipid metabolism, carbon metabolism, and nucleotide metabolism (Figure S17B). Moreover, we confirmed that key purine catabolic enzymes (CD39, CD73, and ADA) were expressed on MCregs and their secreted EVs (Figure 6I). Additionally, we collected cell supernatants from the MCreg-EV-treated and untreated groups for metabolomics. PCA revealed clear differences between the EV and control groups (Figure S17C). A total of 46 differentially abundant metabolites were identified, with 11 upregulated and 35 downregulated (Figure J-K). The differentially altered metabolites were mainly enriched in the urea cycle, purine metabolism, and β-alanine metabolic pathways (Figure 6L).

To explore the role of the purine metabolism enzyme activity of EVs, activated CD4^+^ T cells were treated with MCreg-EVs and AMP (which can be converted to adenosine by CD73). The immune inhibitory effect of EV plus AMP was greater than that of EV alone or AMP alone (Figure 6M). In contrast, the inhibitory effect of MCreg-EVs on CD4^+^ T-cell activation was partially abolished by a CD73 inhibitor (Figure 6N) or an adenosine A2A receptor (A2AR) inhibitor (Figure 6O), suggesting that CD73^high^ MCreg-EVs suppress CD4^+^ T-cell activation by regulating purine catabolism (Figure 6P).

**Figure 7:**
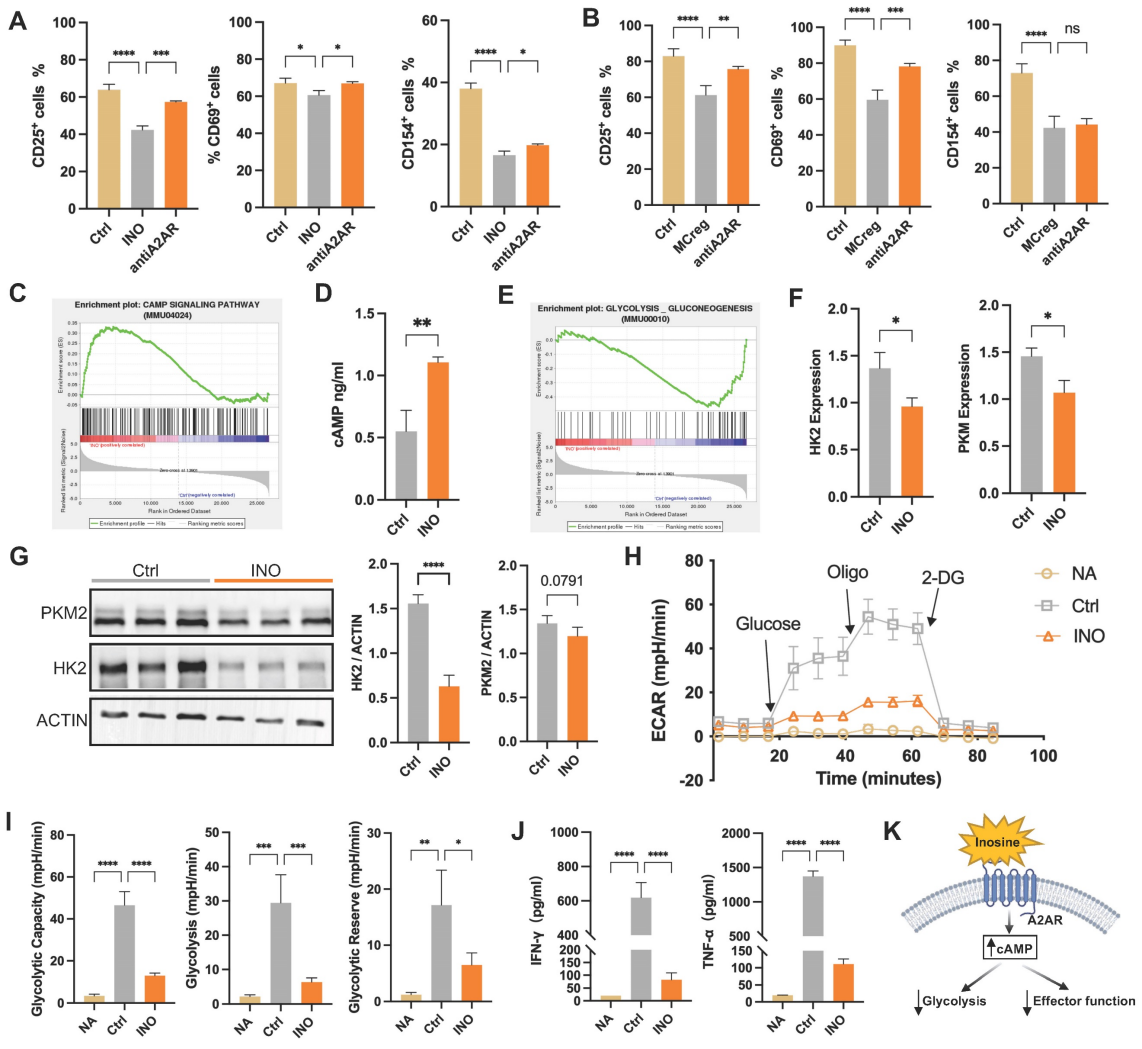
Purine catabolic metabolites activate the cAMP pathway to suppress T-cell activation by inhibiting glycolytic processes. (A) Percentages of CD25^+^, CD69^+^, and CD154^+^ cells among activated CD4^+^ T cells in the control group (Ctrl), inosine-treated group (INO), and A2AR inhibitor-treated group (antiA2AR). (B) Percentages of CD25^+^, CD69^+^, and CD154^+^ cells among activated CD4^+^ T cells in the Ctrl group, iMCreg-treated group (MCreg) and anti-A2AR (A2AR inhibitor) group. (C) Pathway analyses indicating the activation of the cAMP signaling pathway in the INO group. (D) Comparison of the cAMP concentration in the cell supernatant between the Ctrl group and INO group. (E) Pathway analysis indicating the inhibition of glycolysis in the INO group. (F) Assessment of the differences in the transcript expression levels of HK2 and PKM2 between the Ctrl group and INO group. (G) Protein expression analysis of HK2 and PKM2 in the Ctrl group and INO group. (H) ECAR of T cells in the NA group, Ctrl group, and INO group. (I) Glycolytic capacity, glycolytic ability, and glycolytic reserve among the three groups. (J) Concentrations of cytokines released into the cell supernatants. (K) Inosine triggers the cAMP signaling pathway via the A2AR receptor, resulting in the inhibition of glycolytic processes and downstream effector functions in T cells.

The detailed effect of purine catabolic metabolites on T-cell activation was further explored. INO treatment reduced the populations of CD25- and CD154-positive activated CD4^+^ T cells, while this effect was partly attenuated by the A2AR inhibitor, suggesting that INO may activate A2AR to exert an immunosuppressive effect (Figure 7A). Additionally, we found that treatment with A2AR inhibitor (ZM241385) could partially reversed the inhibitory effect of iMCregs on CD4^+^ T-cell activation, which indicates that the immunosuppressive effect of iMCregs is dependent on the A2AR signaling pathway (Figure 7B).

The mechanism underlying INO-mediated T-cell inactivation was explored using RNA-seq. A PCA scatter plot showed a distinct separation between INO-treated CD4^+^ T cells and CD4^+^ T cells alone (Figure S18A), and DEGs between the two groups are listed in Figure S18B and Table S9. GSEA revealed upregulated cAMP pathway activity in CD4^+^ T cells after INO treatment (Figure 7C) and elevated intracellular cAMP concentrations compared to those in the control group (Figure 7D). The cAMP pathway can regulate multiple cellular processes in T cells, such as proliferation, differentiation, and cellular metabolic processes 28. Further GSEA indicated the inhibition of glycolytic processes in INO-treated CD4^+^ T cells (Figure 7E), while it has been reported that enhanced glycolysis is a major pathway for energy supply during T-cell activation 13. Indeed, INO treatment downregulated the expression of vital glycolytic enzymes, including hexokinase 2 (HK2) and pyruvate kinase isoform M2 (PKM2), in activated CD4^+^ T cells (Figure 7F-G). INO treatment reduced the extracellular acidification rate (ECAR), glycolytic capacity, glycolytic ability, and glycolytic reserve of activated CD4^+^ T cells (Figure 7H-I). Compared with activated CD4^+^ T cells, activated CD4^+^ T cells treated with INO showed lower levels of cytokine (IFN-γ and TNF-α) secretion (Figure 7J). Taken together, our results suggest that MCreg-EVs can restrict CD4^+^ T-cell hyperactivation by regulating purine catabolism, thereby enhancing cAMP signaling and suppressing glycolysis (Figure 7K).

## Discussion

Disordered T-cell activation is a critical contributor to chronic liver inflammation, fibrosis and cirrhosis, and recent research underscores the pivotal role of metabolic pathways in modulating immune cell functionality, but the metabolic mechanism governing immune homeostasis after liver injury remains elusive. It has been proposed that the liver can rapidly activate immunity to restore homeostasis after disturbance, and the ultimate outcome of the intrahepatic immune response (e.g., fibrosis or resolution) depends on the balance between proinflammatory and anti-inflammatory cell populations 8. Thus, we hypothesized that certain bone marrow (BM)-derived immune cell subsets may be expanded as a natural response to limit T-cell overactivation during the early phase of liver injury, and such cells may be further developed as specific therapies for restricting T-cell-induced liver inflammation. One of the leading pathological features of the ConA-induced liver injury model is hyperactivation of T cells, particularly CD4^+^ T cells, along with excessive secretion of proinflammatory factors 17.

To explore metabolic changes associated with this process, we profiled metabolic changes in mouse liver/serum samples and human serum samples. The results showed dramatically altered purine nucleotide metabolites, such as ADO and INO, in both ConA mice and AIH patients. The bulk RNA-seq results of mouse liver tissues also indicated that many upregulated or downregulated DEGs in the ConA group were enriched in purine metabolic pathways. According to the bulk metabolomics and transcriptomics results, the bidirectional changes in metabolic genes or metabolites might be due to several possible reasons. First, purine metabolic networks are complex and highly dynamics, which consist of many metabolic loops, branch points and feedback (positive or negative) pathways 29. Second, there are multiple cell types (resident and immune cells) within liver tissues, and each cell type might have a different metabolic pattern even under the same conditions. Nevertheless, these altered metabolites and genes were strongly enriched in pathways related to purine catabolism, suggesting overall altered purine catabolism in response to immune-mediated liver injury. However, the specific mechanism of purinergic signaling in immune-mediated liver injury needs to be further investigated.

Surface nuclear enzymes (e.g., CD39 and CD73) play vital roles in modulating purine catabolism, and the CD39-CD73 axis may function as a critical metabolic checkpoint in various immune disorder-related diseases (e.g., infections, autoimmune diseases and cancer) 20. In our study, the changes in the expression of CD39 and CD73 observed in the bulk-seq results were not consistent with the public scRNA-seq data, which may be due to the differences in the two technical methods. Bulk RNA sequencing uses tissue or cell populations as the starting material, producing mixtures of different gene expression profiles. Consequently, the average gene expression profile obtained may mask the true signals driving biological processes 30. To address this issue, we isolated hepatocytes (comprising ~70% of liver cells) and nonparenchymal cells and detected the expression of CD39 and CD73 separately. We found that CD39/CD73 expression in hepatocytes was consistent with the bulk RNA-seq results, whereas CD39/CD73 expression in nonparenchymal cells showed the opposite trend. In fact, several studies have also shown different expression patterns of CD39 and CD73 in diverse cell subpopulations during disease progression, such as ischemia-reperfusion injury and lung cancer 31, 32.

To elucidate the expression patterns of CD39 and CD73 in liver nonparenchymal cells, we analyzed scRNA-seq data from liver tissues of ConA and NC mice. scRNA-seq has become a highly popular genomic tool for profiling transcriptome heterogeneity, enabling the identification of rare cell types and states by capturing individual cellular transcriptome changes 30. Our results revealed a substantial increase in the proportions of T cells, macrophages, and MCs compared to those in NC mice, and the CD39 and CD73 genes were predominantly expressed on the three cell types. In addition, elevated CD39/CD73 expression in immune cells, such as monocytes, neutrophils and T cells, has been found during inflammation in response to tissue injury 29. Further FCA revealed that CD11b^+^Gr-1^+^ MCs had markedly greater CD39 and CD73 expression than T cells or macrophages, demonstrating the expansion of a CD39^+^CD73^+^Gr-1^high^CD11b^+^ MC subset at an early stage after ConA-induced liver injury. Moreover, the proportions of these MC subsets were negatively correlated with the degree of liver injury in ConA mice, suggesting that these MC subsets might exert a liver-protective effect.

Previous studies have reported the existence of immunosuppressive MC subsets (also expressing CD39/CD73) in the tumor microenvironment 33, but their role in inflammatory liver diseases is poorly understood. The suppressive MC subsets may exert immunoregulatory effects by upregulating enzymes such as IDO, ARG1, and iNOS 34, 35. In line with these reports, we found that MCreg treatment suppressed CD4^+^ T-cell activation and liver injury both *in vivo* and *in vitro* and that CD73^high^ MCregs had greater immunosuppressive effects. Based on these findings, we named these CD39^+^CD73^+^Gr-1^high^CD11b^+^ MCs regulatory MCs (MCregs), and the expansion of MCregs is likely a natural immune response that aims to restrict T-cell hyperactivation at the early stage after liver injury.

Immune cells can impact other cell functions through direct (cell-to-cell attachment) or paracrine (release of cytokines or EVs) mechanisms 12, 27. As cell-derived nanoscale bilayer vesicles, EVs can transfer various bioactive cargos, such as proteins, RNAs, lipids and metabolites, to facilitate intercellular communication *in vivo* 36. It has been reported that suppressive MCs can release EVs to exert immunoregulatory effects in the tumor microenvironment and to mitigate hepatocyte mitochondrial damage in ConA-induced liver injury 37, 38. Similarly, we found that MCreg-derived EVs (MCreg-EVs) can suppress CD4^+^ T-cell activation *in vitro*. In addition to EVs, EVs also carry extracellular AMP catabolic enzymes, such as CD73 39, and CD73^+^ MSC-EVs promote the M2 polarization of macrophages to reduce inflammation in spinal cord injury by converting extracellular ATP to adenosine 40. Additionally, we found that MCreg-derived EVs expressed critical purine catabolic enzymes, including CD39, CD73, and ADA. Conversely, the immunosuppressive effect of MCreg-EVs on activated CD4^+^ T cells decreased after blocking CD73 or A2AR, a G protein-coupled receptor responsive to ADO 41.

Purine metabolism byproducts (INO and ADO) are recognized for their immunomodulatory properties 42. We confirmed that ADO or INO treatment alleviated CD4^+^ T-cell activation in ConA-induced liver injury. We further observed upregulated cAMP signaling but suppressed glycolytic processes in INO-treated CD4^+^ T cells, and activation of A2AR by ADO was shown to stimulate the cAMP pathway to affect T-cell function 28. cAMP signaling may inhibit the glycolytic pathway by activating the downstream protein kinase A (PKA) pathway 28, which further impacts the activity of glycolytic enzymes, such as HK2 and PKM2 43, 44. Taken together, our findings suggest that MCregs suppress CD4^+^ T-cell hyperactivation at least partly via CD73^high^ EV-mediated metabolic regulation.

To date, there has been growing interest in the use of immunosuppressive MCs for cellular therapy for transplantation and treatment of autoimmunity 45. For example, studies in human allogeneic hematopoietic stem cell transplantation (allo-HSCT) have shown that the infusion of these MCs (HLA-DR^-/low^CD33^+^CD16^-^) alleviated acute graft-versus-host disease (GVHD) in a humanized murine model, with the number of MCs inversely correlated with the incidence of GVHD in patients 46. Additionally, Park *et al*. successfully generated suppressive MCs (CD11b- and CD33-positive) *in vitro* from human cord blood units for large-scale production, demonstrating strong inhibition of T-cell proliferation and increased anti-inflammatory cytokine production 47. However, further studies are needed to assess the *in vivo* function and safety of these MCs. Moreover, adoptive transfer of these MCs has been explored in murine models of various autoimmune diseases, such as autoimmune arthritis and multiple sclerosis 48, 49. Although sufficient clinical evidence for MC-based cell therapy is lacking at present, preclinical models suggest promising outcomes. The expansion of the regulatory MC subset is likely a natural response that restricts excessive liver inflammation, and adoptive transfer or *in vivo* induction of MCregs may be a potent strategy for mitigating T-cell hyperactivation and T-cell-mediated liver injury.

Notably, the therapeutic effect of MCregs or MCreg-EVs on inhibiting T-cell hyperactivation is promising, but some incompletely understood questions need to be answered in future studies. First, we were unable to definitively ascertain the specific contribution of CD39^+^CD73^+^Gr-1^high^CD11b^+^ MCs to overall purine metabolism in injured liver tissues. Although we generated MCregs with highly similar characteristics *in vitro* and demonstrated their immunomodulatory role through the CD73 axis, we could not determine the precise contribution of this MC subset to the total ADO in the injured liver. Second, the immune suppression of MCregs might also be facilitated by other purine enzymes, such as CD39 and ADA, both of which are detectable in MCregs and their released EVs. Despite the effectiveness of targeting the purine catabolic axis regardless of specific purine enzymes, our future studies will delve into the functions of other enzymes influencing purine metabolism. Finally, while human and mouse MCregs share similarities as heterogeneous populations of immature MCs, there may be nuanced differences 26. Although the ConA-induced mouse model has proven valuable in mirroring human AIH in preclinical studies 50, the insights gained from this study merely offer preliminary indications for clinical combination therapy. Thus, further exploration and validation are imperative in future studies.

## Materials and Methods

### Immune-mediated Mouse Hepatitis Model

The animal experiments were approved by the Animal Ethical and Welfare Committee of West China Hospital of Sichuan University (No. 20220304043). C57BL/6 mice, aged 6-8 weeks and weighing 20-22 g, were utilized to induce ConA-induced liver injury. The mice were housed in a standard experimental environment with protocols approved by the Animal Ethical and Welfare Committee of West China Hospital of Sichuan University and received a single 10 mg/kg body weight injection of ConA (Sigma, USA) in 200 μL of phosphate-buffered saline (PBS, Servicebio, China) via the tail vein 38. After sacrifice at 12 h, 24 h, 36 h, and 48 h post-ConA injection, samples were collected for ALT and AST level determination and hepatic T-cell activation assessment at different time points.

### Patient Enrollment and Sample Collection

This study was approved by the Ethics Committee of West China Hospital of Sichuan University (No. 2013-221), and written informed consent was obtained from each patient. This study included 73 peripheral blood samples from AIH patients diagnosed at West China Hospital between January 2019 and November 2022, following International Autoimmune Hepatitis Group (1999) guidelines 51, 52. The clinical data are shown in Table S10. Patients who received standard therapy were divided into complete response (CR) and nonresponse (NR) groups based on 6-month aminotransferase levels 53. The complete response was defined as a normalization of both transaminase and IgG concentrations within 12 months after treatment initiation 54. Patients with normalized transaminases and IgG levels were defined as responders, while patients who did not meet these criteria were categorized as nonresponders. Metabolomic analysis was performed on plasma samples randomly selected from 48 AIH patients and 36 healthy controls. The entire study received approval from the Ethics Committee of West China Hospital and adhered to relevant regulations.

### LC-Mass Spectrometry (LC‒MS)-based Metabolomic Analysis

After centrifugation at 2000 g for 10 min, the serum samples were transferred to Eppendorf tubes and resuspended in 80% methanol. After vortexing and a 5 min ice incubation, the samples were centrifuged at 15000 g for 20 min. The clarified samples were then transferred and subjected to an additional centrifugation at 15000 g for 20 min before LC/MS injection. Novogene Co., Ltd. (Beijing, China) conducted the study. To identify and annotate the detected metabolites, we utilized the KEGG (http://www.genome.jp/kegg/), HMDB (http://www.hmdb.ca/), and Lipidmaps (http://www.lipidmaps.org/) databases. Both principal component analysis (PCA) and partial least squares discriminant analysis (PLS-DA) were conducted using metaX. Metabolites with a VIP score > 1.0 and a p value < 0.05 were considered to be differentially abundant. Further functional insights and pathway analyses were performed using the KEGG database.

### Transcriptome Sequencing, Annotation and Bioinformatics

RNA sequencing was performed by Novogene Co., Ltd. (Beijing, China) using the Illumina HiSeq 3000 platform. Differentially expressed (DE) mRNAs were identified with a q-value (false discovery rate) < 0.05 and an absolute log2-fold change (|log2FC|) ≥ 1. Functional analysis of DEmRNAs involved in Gene Ontology (GO) analysis (http://www.geneontology.org) and KEGG pathway analysis. Significantly enriched GO terms and KEGG pathways were determined with corrected p values < 0.05, providing valuable insights into the functional implications of the identified DEGs and their involvement in various biological processes.

### Analysis of Liver Tissue scRNA-seq Data from ConA Mice

The scRNA-seq data and raw count matrices were obtained from the Gene Expression Omnibus (GEO) database of the National Center for Biotechnology Information (NCBI) (https://www.ncbi.nlm.nih.gov/geo/). The GEO accession number is GSE201006, which comprises transcriptional profiling of live tissue from ConA and NC mice, as published by Cannon AS *et al* 21. Differential gene expression analysis was performed to identify marker genes for distinct cell clusters using several approaches to enhance cluster subtype determination. Using the FindAllMarkers function within the Seurat R package, we identified representative markers for each cluster. Furthermore, we assessed the expression of CD39 and CD73 across different cell types and examined differential changes in both the NC and ConA groups.

### Flow Cytometric Analysis and Sorting

Liver nonparenchymal cells were isolated using Percoll (Solarbio, China) and suspended in PBS 19. Peripheral blood mononuclear cells from patients were obtained using human lymphocyte separation medium (Dakewei, China) and washed with PBS. Approximately 1 × 10^6^ cells were suspended in 100 μL of PBS and stained with fluorochrome-coupled antibodies at 4 °C for 30 min. After staining, the cells were washed twice with PBS. Flow cytometry was performed with an LSRFortessa flow cytometer (BD Bioscience, USA) or a CytoFLEX flow cytometer (Beckman, USA). Data analysis was performed using FlowJo software. The following antibodies were obtained from BioLegend (USA): anti-mouse CD45-AF750, anti-CD4-TITC, anti-CD8-Percp-Cyanine 5.5, anti-CD25-PE, anti-CD69-APC, anti-CD154-PE-Cyanine 7, anti-CD11b-APC, anti-Gr1-FITC, anti-CD73-PE, anti-CD39-PE-Cyanine 7, anti-F4-80-Percp-Cyanine 5.5, anti-Gr1-PE-Cyanine 7, anti-Ly6g-FITC, and anti-Ly6c-PE; and anti-human CD33-FITC, anti-CD14-PE, anti-CD15-Percp-Cyanine 5.5, HLA-DR-PE-Cyanine 7, anti-CD11b-FITC, anti-F4/80-PE, anti-CD206-APC, anti-CD86-PE-Cyanine 7, anti-Foxp3-AF750 and anti-DAPI-PB450. Cell sorting was performed using a FACSAria III cell sorter (BD Bioscience). For induced MCregs, the gating strategy included the identification of CD11b^+^Gr1^+^ cells for conventional MCregs, CD39 high-expressing cells for CD39^high^ MCregs, CD73 high-expressing cells for CD73^high^ MCregs.

### Isolation and Induction of MCregs

MCregs were induced following established procedures 24. Specifically, leucocytes derived from C57BL/6 mouse bone marrow were cultured in 2 × 10^6^ / 100 mm dishes in 10 mL of RPMI 1640 medium supplemented with GM-CSF (10 ng/mL, Novoprotein, China), IL-6 (10 ng/mL, Novoprotein, China), 50 μM β-mercaptoethanol (Sigma-Aldrich, USA), 1 mM sodium pyruvate (Gibco, USA), 2 mM L-glutamine, 100 μg/mL streptomycin, 100 U/mL penicillin, and 10% FBS. After 3 days, 10 mL of complete medium containing the aforementioned substances was added to the dish, and the cells were harvested on day 4 for subsequent experiments. Induced MCregs were primarily identified by the proportion of CD11b^+^Gr1^+^ cells, their ability to release ROS and NO, and the expression levels of the signature genes NOX2, NOS2, ARG1, and PD-L1. Their immunosuppressive capacity was assessed mainly by coculturing CD4^+^ T cells and measuring changes in the activation indices of CD4^+^ T cells using flow cytometry.

### Animal Experimental Designs

In the adoptive transfer of MCregs experiment, all mice were randomly divided into the NC group, ConA group and MCreg-treated group (ConA+M). Subsequently, 5 × 10^6^ MCregs were intravenously injected 30 min before ConA administration. For CD73^high^ MCreg treatment, cells were sorted by flow cytometry and then adoptively transferred into ConA-induced mice. The mice were randomly divided into the ConA group, conventional MCreg-treated group (Con.M+ConA), and CD73^high^ MCreg-treated group (CD73^high^.M+ConA). In the metabolite administration experiment, the mice received intravenous injections of 200 mg/kg ADO or 300 mg/kg INO 30 min before ConA injection. All mice were sacrificed at 24 h after ConA administration, and samples were collected for analysis.

### Isolation of MCreg-derived EVs

To collect EVs released by MCregs, the cells were cultured in EV-depleted medium for an additional 48 h. The medium was then subjected to a modified differential ultracentrifugation method 55: 300 g for 10 min, 1000 g for 15 min, and 10000 g for 30 min, followed by ultracentrifugation at 100000 g for 90 min using an SW32Ti rotor in an Optima XPN-100 (Beckman Coulter, USA). The resulting pellets were washed with PBS and centrifuged at 100000 g for 90 min at 4 °C. The purified EVs were resuspended in PBS for further analyses.

### Nanoparticle Tracking Analysis (NTA)

We employed nanoparticle tracking analysis (NTA) using the Zeta View PMX 120 system from Particle Metrix, Germany, to measure the size and quantity of the EVs. The EVs were diluted 1000 times with ultrapure water. The specified parameters for the analysis were as follows: sensitivity: 80, shutter: 100, minimum brightness: 30, minimum area: 10, and maximum area: 1000. All analyses were conducted at a temperature of 25 °C.

### Transmission Electron Microscopy (TEM)

For the observation of EV morphology, negative staining was conducted. Diluted EVs were applied to copper grids, stained with 2% phospho-tungstic acid, and subsequently examined using transmission electron microscopy (H-600, Hitachi, Japan) at 75 kV.

### Proteomic Analysis

MCreg-EVs from a -80 °C freezer were concentrated, mixed with 8 M urea lysis buffer, vortexed, ultrasonically dissolved, incubated on ice, and centrifuged, after which the supernatant was collected for protein quantification. For trypsin digestion, protein solutions were treated with DTT and IAM, diluted with Tris-HCl, and digested with trypsin at a 1:100 ratio for 16 h at 37 °C, followed by peptide desalting and freeze-drying. LC‒MS analysis involved redissolving peptide powder in 0.1% formic acid using an EASY-nLC 1200 ultrahigh-performance liquid chromatography system for elution. Mobile phase A was 0.1% aqueous formic acid, and mobile phase B was 0.1% formic acid in acetonitrile, following a specific elution gradient. The ionized peptides were analyzed with a Thermo Scientific Q ExactiveTM HF-X mass spectrometer in DIA mode (Novogene Co., Ltd., China) with high-resolution detection. The secondary mass spectrometry data were searched using DIA-NN (v1.8.1) with specific parameters. GO and KEGG were used to analyze the protein families and pathways, respectively.

### Western Blotting Analysis

Total protein was extracted from EVs and cells with radioimmunoprecipitation assay buffer (CWBIO, China) supplemented with protease and phosphatase inhibitors (Selleckchem, USA). Proteins (minimum 10 μg) were separated through sodium dodecyl sulfate-polyacrylamide gel electrophoresis, followed by transfer to polyvinylidene difluoride membranes (Merck Millipore). After a 1.5 h block with 5% BSA, the membranes were incubated with primary antibodies, including anti-HSP70 (HuaBio, China), anti-TSG101 (HuaBio, China), anti-cytochrome C (HuaBio, China), anti-Alix (HuaBio, China), anti-CD39 (HuaBio, China), anti-CD73 (HuaBio, China), anti-ADA (Immunoway, China), anti-ACTIN (Cell Signaling Technology, USA), anti-PKM2 (Abcam, USA) and HK2 (Abcam, USA), at 4 °C overnight, followed by secondary antibody treatment (HRP-conjugated, Proteintech, China) for 1 h at room temperature. Band detection was performed with an ultrahigh-sensitivity ECL kit (MedChemExpress, USA), and ImageJ software was used to quantify expression levels relative to actin expression.

### Distribution of MCregs in ConA-Induced Mice

MCregs were labeled with CM-Dil (Yeasen, China) following the manufacturer's instructions. In brief, the cells were incubated in the staining working solution at 37 °C for 5 min and then at 4 °C for 15 min, followed by washing with PBS. The labeling efficiency and cell morphology were examined using fluorescence microscopy (Leica, Germany). After labeling, the cells were intravenously injected into ConA mice via the tail vein. After 24 h, liver tissue sections were stained with DAPI.

### Histology, Immunofluorescence, and TUNEL Assay

For histological examination, livers fixed in 10% formalin were paraffin-embedded and sectioned into 5 μm slices. Hematoxylin and eosin (H&E) staining (Servicebio, Ltd., China) was performed on the sections. The histological scores of six randomly selected fields per section were assessed following Suzuki's criteria for liver injury 56. Immunofluorescence was performed utilizing CD11b, CD4, F4/80, CD206, and iNOS antibodies (Abcam, USA), and TUNEL staining was performed with a TUNEL BrightRed Apoptosis Detection Kit (Biossci biotech Co., Ltd., China). The average number of TUNEL^+^ cells in six fields per section was used to determine the apoptotic cell ratio. Evaluation was performed in a double-blinded manner by at least three expert researchers.

### Assessment of Liver Function

We assessed liver function and injury by measuring serum alanine aminotransferase (ALT) and aspartate aminotransferase (AST) levels using an automatic dry biochemical analyzer (Hitachi, Japan).

### Quantitative Real-time PCR (RT‒qPCR) Analysis

Total RNA was extracted from liver tissues and cells in each group using TRIzol reagent (Tiangen, China). RNA quality was evaluated with an Agilent 2100 Bioanalyzer (Agilent Technologies, USA) and a Qubit Fluorometer (Invitrogen). cDNA synthesis was achieved using a PrimeScript RT reagent kit (Takara, Japan), and all primers were obtained from Tsingke (Beijing, China). RT‒qPCR was performed using SYBR Green Supermix on a CFX96 RT‒qPCR detection system (Bio-Rad, USA). The expression levels of target genes were normalized to the actin expression level.

### Preparation and Coculture of Mouse CD4^+^ T Cells

Mouse CD4^+^ T cells, which were isolated from the spleen via a MojoSort™ Mouse CD4^+^ T-cell isolation kit (BioLegend, USA), exhibited > 95% purity. After being cultured in RMPI 1640 medium supplemented with recombinant murine IL-2 (20 IU/mL, Novoprotein, China), the cells were stimulated with plate-bound anti-CD3 (5 μg/mL, Biolegend, USA), soluble anti-CD28 (5 μg/mL, Biolegend, USA), or PMA (10 ng/mL, MultiSciences, China). In coculture, CD4^+^ T cells (5 × 10^5^ cells / well) were paired with MCregs at 1:1, 2:1, 4:1, and 8:1 ratios. In additional experiments, ADO (5 mM, MedChemExpress, USA), INO (5 nM, MedChemExpress, USA), AMP (10 μM, MedChemExpress, USA), or 1 × 10^10^ MCreg-EVs were individually added. After 24 h (anti-CD3/anti-CD28) or 6 h (PMA) of stimulation, the expression of the activation markers CD25, CD69, and CD154 on the surface of CD4^+^ T cells was assessed by flow cytometry.

### Enzyme-linked Immunosorbent Assay (ELISA)

The levels of IFN-γ and TNF-α were quantified with mouse ELISA detection kits following the manufacturer's guidelines (Dakewei, China & MultiSciences, China). cAMP levels in the cell supernatant were measured according to the manufacturer's instructions (Abcam, USA). The optical density values were recorded at 450 or 570 nm using a microplate reader (BioTek, USA).

### Intracellular NO Production Assays

The induced MCregs were treated as indicated, washed three times with PBS and lysed with lysis buffer. NO levels in the supernatants were measured according to the manufacturer's instructions (Beyotime, China).

### Measurement of the Extracellular Acidification Rate (ECAR)

The ECAR was assessed using a Seahorse XFe24 Flux Analyzer (Seahorse Bioscience, Agilent). Briefly, 5 × 10^5^ cells were seeded in a 96-well XF Seahorse incubation plate according to the manufacturer's protocol. Cells were cultured at 37 °C in XF base medium, and glucose (10 mM), glutamine (1 mM), 2-DG (50 mM) and oligomycin (1 μM) were added sequentially to the plates at specific time points following the manufacturer's guidelines. The ECAR was measured and plotted using Seahorse XF24 software.

### Statistical Analysis

The continuous variables are expressed as the mean ± SD. We conducted rigorous statistical analyses employing GraphPad Prism 9 (GraphPad Software, San Diego, CA, USA), SPSS (version 20.0, Chicago, IL, USA), and R software (version 4.1.0). Student's t test or analysis of variance (ANOVA) was utilized for comparisons. Pearson's correlation test was used to evaluate correlations. Statistical significance was denoted as follows: *P < 0.05; ** P < 0.01; *** P < 0.001.

## Supplementary Material

Supplementary figures and tables.
