# Supplementary material for: Engaging natural regulatory myeloid cells to restrict T-cell hyperactivation-induced liver inflammation via extracellular vesicle-mediated purine metabolism regulation
Source: Theranostics. 2024 Aug 12;14(12):4874–93. doi: 10.7150/thno.97427 (PMC11373623; doi:10.7150/thno.97427)
Supplement: Supplementary file 1 — Supplementary figures and tables. [file thnov14p4874s1.zip › Supplementary information/Supplementary information.docx]

**Supplementary information**

**Engaging natural regulatory myeloid cells to restrict T-cell hyperactivation-induced liver inflammation via extracellular vesicle-mediated purine metabolism regulation**

Fan Yang^1#^, Ruoting Men^1#^, Linling Lv^1^, Leyu Zhou^1^, Qiaoyu Deng^1^, Xianglin Wang^1^, Jingping Liu^2^*, Li Yang^1^*

1. Department of Gastroenterology and Hepatology and Laboratory of Gastrointestinal Cancer and Liver Disease, West China Hospital of Sichuan University, Chengdu 610041, China
2. NHC Key Laboratory of Transplant Engineering and Immunology, Frontiers Science Center for Disease-related Molecular Network, West China Hospital of Sichuan University, Chengdu 610041, China

^#^These authors contributed equally to this work and share first authorship

*Corresponding authors:

Li Yang, Professor, M.D.

Email: yangli_hx@scu.edu.cn

Jingping Liu, Professor, M.D.

Email: liujingping@scu.edu.cn

Contacting address:

No. 37^th^ Guoxue Road, Department of Gastroenterology and Hepatology and Sichuan University-University of Oxford Huaxi Joint Centre for Gastrointestinal Cancer, West China Hospital of Sichuan University, Chengdu, Sichuan, 610041, China

**Figures**

**Figure S1**

**Figure S1** Concanavalin A (ConA)-induced mouse model at different time points. (A) Mice were subjected to a single 10 mg/kg body weight injection of ConA via the tail vein and sacrificed at 12 h, 24 h, 36 h, and 48 h post-ConA injection. Tissue samples were collected at each time point for the assessment of aminotransferase levels and hepatic T-cell activation. (B) The plasma levels of alanine aminotransferase (ALT) and aspartate aminotransferase (AST). (C) The percentage of CD25- and CD69-positive CD4^+^CD45^+^ cells. (D) The percentage of CD25- and CD69-positive CD8^+^CD45^+^ cells. (E) H&E staining and histological scoring of liver tissues at 12 h and 24 h after ConA injection (scale bar = 1000 µm).

**Figure S2**

**
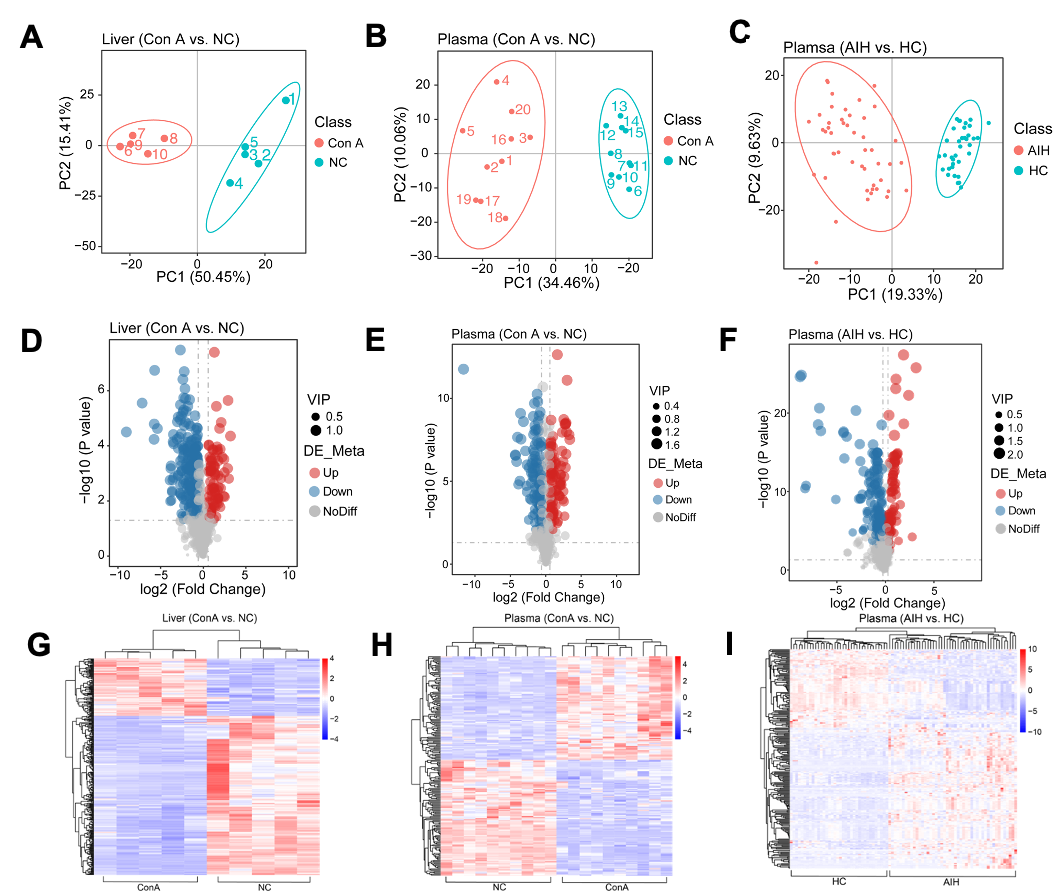
**

**Figure S2** Liver and plasma metabolic profiles of mice from the ConA and NC groups and plasma metabolic profiles of humans from AIH patients and healthy controls (HCs). (A-C) Principal component analysis (PCA) plots between the ConA and NC groups and between the AIH and HC groups. (D-F) Volcano maps showing the differentially abundant metabolites between these groups. (G-I) Unsupervised hierarchical clustering plots based on the differentially abundant metabolites.

**Figure S3**

**
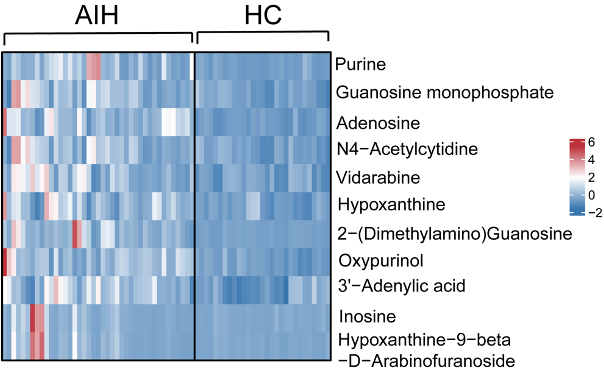
**

**Figure S3** The expression patterns of differentially expressed purine nucleotides between the AIH and HC groups.

**Figure S4**

**
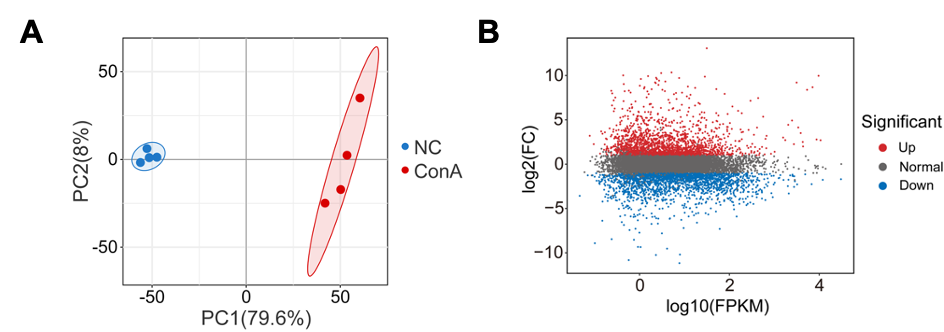
**

**Figure S4** Bulk RNA-seq analysis of liver tissues from ConA and NC mice. The PCA scatter plot (A) and volcano map (B) between the ConA and NC groups.

**Figure S5**

**Figure S5** Detection of CD39 and CD73 expression in isolated mouse hepatocytes and nonparenchymal cells. (A) Mouse hepatocytes and nonparenchymal cells were collected for detection of CD39 and CD73 expression. (B) The CD39 and CD73 expression in hepatocytes between NC and ConA mice. (C) The CD39 and CD73 expression in nonparenchymal cells between NC and ConA mice.

**Figure S6**


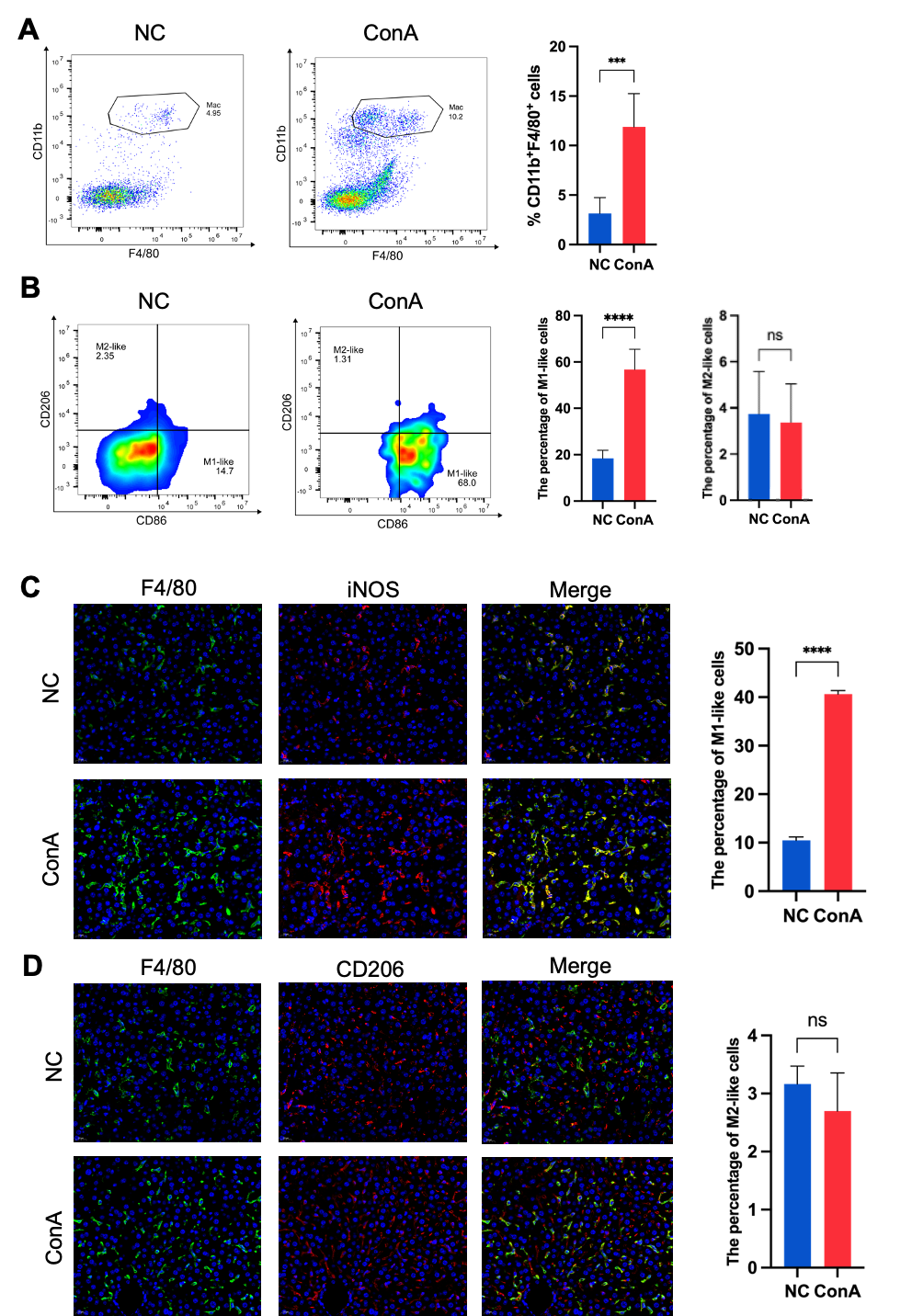


**Figure S6** Detection of liver macrophage subpopulations in the ConA-induced liver injury. (A-B) The percentages of CD11b^+^F4/80^+^ macrophages, CD86^+^ M1-like macrophages, and CD206^+^ M2-like macrophages between groups (n = 5-7). (C-D) Immunofluorescence staining showed F4/80 and iNOS double-positive M1-like cells and F4/80 and CD206 double-positive M2-like cells in mouse livers (scale bar = 20 µm).

**Figure S7**


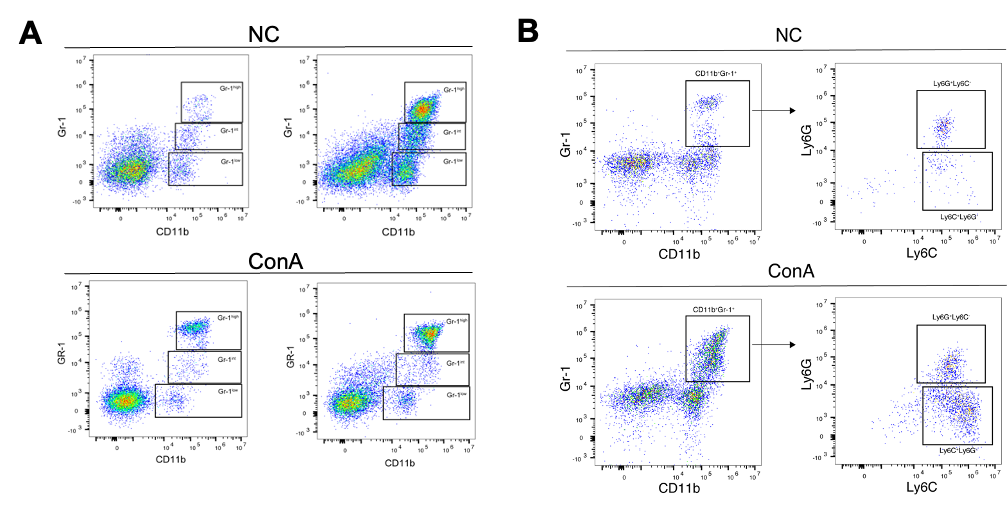


**Figure S7** Representative flow cytometry plots of CD11b^+^Gr-1^+^ myeloid cells (MCs) (A) and granulocytic and monocytic MC lineages (B) between the ConA and NC groups.

**Figure S8**

**
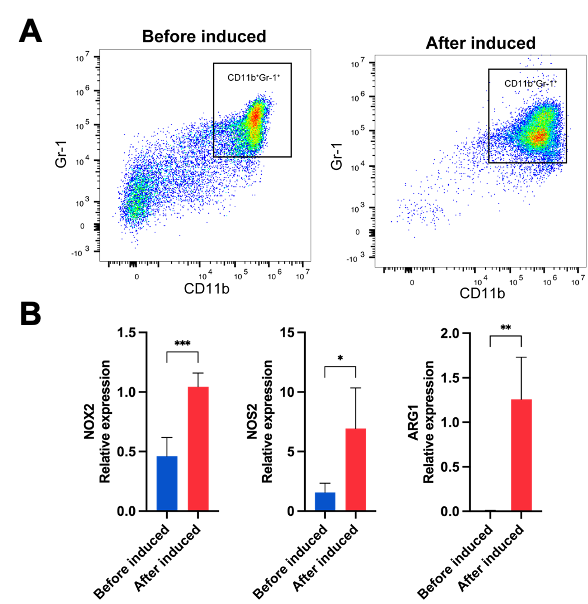
**

**Figure S8** (A) The expression levels of Gr-1 on the induced MCs. (B) The relative expression of NOX2, NOS2 and ARG1 on the MCs before and after induction.

**Figure S9**


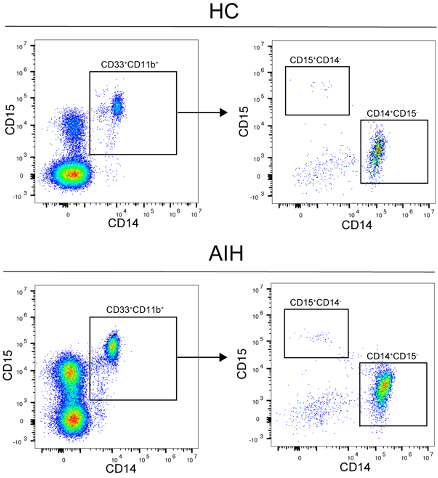


**Figure S9** Representative FCA plots of circulating MCs between the AIH and HC groups.

**Figure S10**


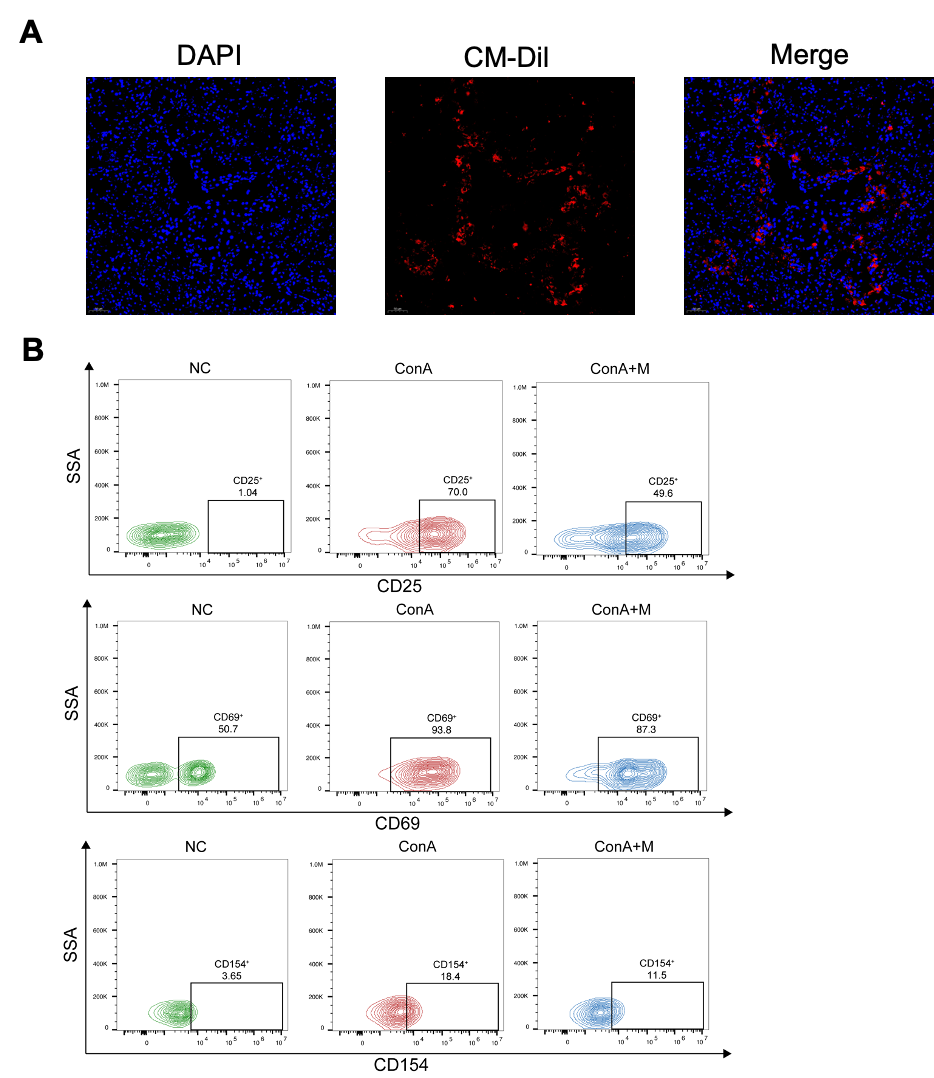


**Figure S10** Adoptive transfer of regulatory MCs (MCregs) into ConA-induced mice. (A) The distribution of MCregs in mouse liver tissues 24 h after i.v. injection. Red represents CM-Dil-labeled MCregs. (B) Representative FCA plots of CD25-, CD69- and CD154-positive CD4^+^ T cells among the NC, ConA-treated and MCreg-treated groups.

**Figure S11**

**Figure S11** Bulk RNA-seq of livers from ConA-induced and MCreg-treated mice. (A) The PCA scatter plot. (B) KEGG pathway analysis of the downregulated DEGs. (C-E) GSEA of the downregulated DEGs.

**Figure S12**

**Figure S12** Liver and plasma metabolic profiles of mice in the MCreg-treated (ConA+M) and ConA groups. (A-B) PCA scatter plots. (C) Volcano plot showing the differentially abundant metabolites in the plasma between the ConA+M and ConA groups. (D) The expression pattern of differentially expressed purine nucleotides between the ConA+M and ConA groups. (E) The serum levels of adenosine and inosine in the ConA+M and ConA groups. (F) Comparison of blood adenosine deaminase (ADA) activity between the two groups.

**Figure S13**

**Figure S13** (A) Representative flow cytometry plots of CD25- and CD69-positive CD4^+^CD45^+^ cells among the NC, ConA- and inosine-treated groups. (B) The relative percentage of live CD4+ T cells treated with adenosine (ADO) or inosine (INO).

**Figure S14**

**
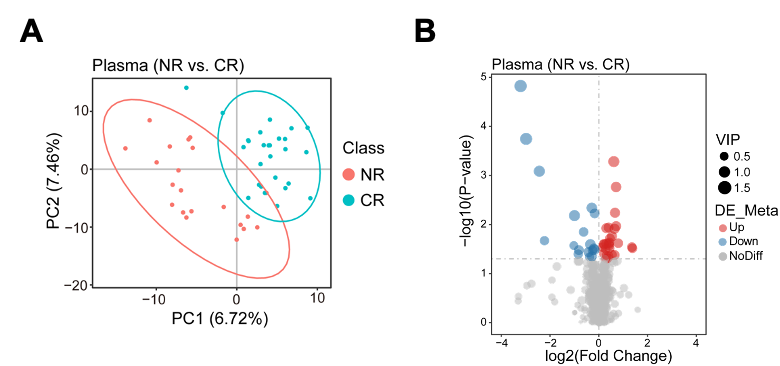
**

**Figure S14** Plasma metabolic profiles of patients with AIH who achieved a complete response (CR) or no response (NR). The PCA scatter plot (A) and volcano map (B) between the CR and NR groups.

**Figure S15**

**Figure S15** CD39^high^ and CD73^high^ MCregs were flow-sorted from total MCregs. (A) Flow cytometry (FCA) sorting gating strategy. (B) Representative FCA plots of CD25- and CD154-positive cells in the Ctrl group, Conventional (Con.) M group, and CD39^high^.M group, and CD73^high^.M group. (C) Representative FCA plots of CD25- and CD154-positive cells in the ConA group, Con.M+ConA group, and CD73^high^.M+ConA group.

**Figure S16**

**
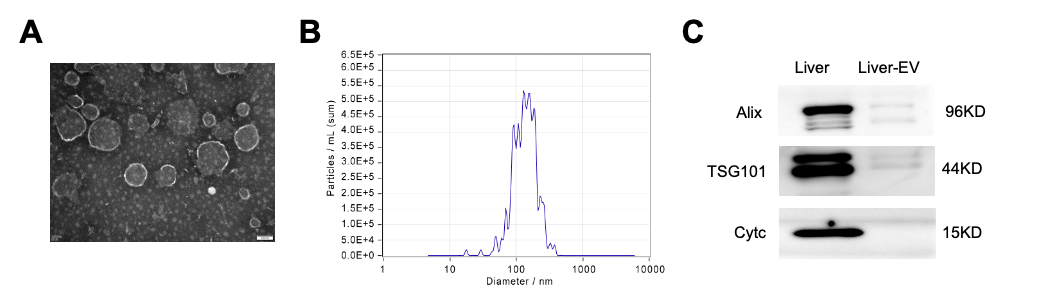
**

**Figure S16** The identification of liver-derived EVs from ConA and NC mice. (A-B) TEM (scale bar = 100 nm) and NTA images of liver tissue-derived EVs. (C) Assessment of the expression levels of EV marker proteins (Alix, TSG01, and Cytc).

**Figure S17**

**Figure S17** (A) The percentages of CD25^+^Foxp3^+^ Tregs in the Ctrl and EV groups. (B) KEGG pathway analysis of the proteins of MCreg-EVs. (C) PCA scatter plot of the plasma metabolic profile between the Ctrl and EV groups.

**Figure S18**


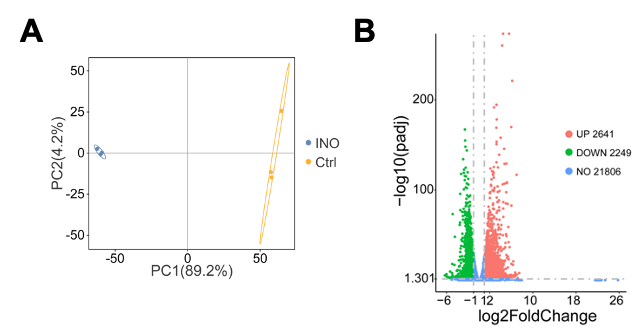


**Figure S18** Bulk RNA-seq analysis of cells from the control (Ctrl) and inosine-treated (INO) groups. The PCA scatter plot (A) and volcano map (B) between the Ctrl and INO groups

**Table S1. Differentially metabolites in liver samples between ConA and NC mice.**

| Name | log2FC | P value |
| --- | --- | --- |
| N-Acetylhistamine | 1.29777843 | 4.05E-08 |
| Lactose | -9.0155794 | 3.14E-05 |
| Trigonelline | -2.6858098 | 1.12E-05 |
| Uridine | -5.3803727 | 2.36E-05 |
| NG,NG-Dimethyl-L-arginine | -2.2334567 | 2.02E-07 |
| Sucrose | -5.7509966 | 1.61E-05 |
| SDMA | -2.0800995 | 1.91E-06 |
| dUMP | -1.6035139 | 2.12E-06 |
| 5-Hydroxymethyluracil | 3.18939859 | 4.42E-05 |
| alpha-D-Glucose | -2.3047881 | 5.02E-06 |
| N-Acetylmannosamine | -2.1500469 | 8.43E-07 |
| Furfural | -3.0727522 | 5.73E-05 |
| L-Homocitrulline | 2.90002695 | 2.26E-06 |
| 2,3-Dinor-TXB2 | -7.1651638 | 2.81E-06 |
| Trehalose | -5.7701323 | 5.81E-05 |
| D-Galactose | -2.3792566 | 5.92E-06 |
| thiamine phosphate | -2.0858889 | 1.38E-05 |
| Fructose | -2.9348708 | 9.03E-06 |
| Ethambutol | -1.8206384 | 4.93E-05 |
| Acetylcholine | -2.3024027 | 0.00022202 |
| Imidazoleacetic acid | -2.8105328 | 4.91E-05 |
| Sorbitol | -2.5682607 | 8.15E-06 |
| 1-Methylnicotinamide | -3.0419026 | 1.34E-05 |
| Hypotaurine | 2.10414098 | 1.41E-05 |
| L-Arabinose | -1.8076604 | 7.28E-05 |
| Pyrophosphate | -0.8829061 | 7.35E-05 |
| 2-Aminoethylphosphonate | -2.2429554 | 1.85E-05 |
| Cyclohexaneacetic acid | 2.86033086 | 0.0006606 |
| 1,4-Diaminobutane | 1.90553011 | 2.34E-05 |
| Trimethylamine N-oxide | -3.5116575 | 3.86E-05 |
| N4-Acetylcytidine | 1.63703605 | 0.00016068 |
| Allopurinol | -3.5448461 | 7.11E-05 |
| 2-Hydroxycinnamate | -0.7391215 | 2.19E-05 |
| Deoxyribose 5-Phosphate | 0.91891714 | 0.00077106 |
| 3-Methylxanthine | -0.8736116 | 2.68E-05 |
| Inosine | -2.583169 | 3.67E-05 |
| O-Acetyl-L-homoserine | -0.7398802 | 5.26E-05 |
| Creatine phosphate | 0.98767578 | 0.00034102 |
| N-Acetylthreonine | -0.8736411 | 0.00074599 |
| 3-Methylindole | -0.881645 | 5.53E-05 |
| Thymidine 3,5-cyclic monophosphate | -2.2406323 | 0.00013303 |
| L-Hydroxylysine | -2.3234626 | 0.00014287 |
| Isonicotinic acid | -1.4609675 | 6.94E-05 |
| Allantoin | 1.9924837 | 0.00013229 |
| 1-Methylxanthine | 2.21831745 | 0.00025665 |
| Octopamine | 1.61281949 | 0.00010776 |
| 5beta-Androstan-17beta-ol-3-one | -1.7991811 | 0.00016418 |
| Isoquinoline | -0.9637252 | 0.00012377 |
| 5-Hydroxyindoleacetate | 2.43970097 | 0.00030607 |
| 2-(Dimethylamino)Guanosine | 1.27535508 | 0.00053949 |
| N-Acetylglucosamine 1-phosphate | -2.5646233 | 0.00081266 |
| N-Formyl-L-methionine | 0.93853208 | 0.00029884 |
| 1-Methylguanosine | 1.17482674 | 0.00014283 |
| 1-(4-Methoxyphenyl)-2-propanone | -0.669984 | 0.00010206 |
| Taurine | -0.626878 | 0.00010343 |
| N7-Methylguanosine | 1.08007854 | 0.00013445 |
| L-Leucyl-L-Alanine | -2.3048949 | 0.00011778 |
| Dulcitol | -1.8216417 | 0.0001161 |
| Trehalose 6-phosphate | -2.9374306 | 0.00012363 |
| 4-Pregnen-17alpha,20alpha-Diol-3-One | -1.2094688 | 0.00013723 |
| 4-Amino-5-imidazolecarboxamide | -0.8688349 | 0.0001286 |
| Octanedioic acid | -1.9784052 | 0.00113903 |
| 2-Aminooctanoic acid | -0.6699228 | 0.00025647 |
| Gluconolactone | -1.3876571 | 0.00033792 |
| L-Carnitine | -0.6648937 | 0.00200037 |
| L-Tryptophan | -1.0287796 | 0.00020862 |
| 5-Methoxyindole-3-acetic acid | -0.7275434 | 0.00023037 |
| L-Hydroxyproline | -0.5937812 | 0.00054317 |
| Nicotinamide | -0.7633542 | 0.00024094 |
| Hippuric acid | -2.0647479 | 0.00058271 |
| Glutarylcarnitine | -1.7130933 | 0.00074895 |
| L-Gulono-1,4-lactone | -0.9483907 | 0.00056691 |
| Xanthosine | 0.61872401 | 0.00081809 |
| Tricarballylic acid | -2.9616365 | 0.00121517 |
| allantoate | 1.75813534 | 0.00035045 |
| Propionylcholine | -0.5338014 | 0.00121301 |
| 4-Pyridoxic acid | -1.1091046 | 0.00036821 |
| L-Canavanine | 0.90589051 | 0.00082573 |
| Serotonin | 0.90832654 | 0.00057252 |
| Phe-Phe | -1.3095329 | 0.0002522 |
| Picolinic acid | -0.9736876 | 0.00037201 |
| Cyclohexylsulfamate | -1.1246161 | 0.00067923 |
| 2-Methylpentanedioic acid | -2.6102501 | 0.00051487 |
| L-Arabinitol | -0.9298683 | 0.00030173 |
| Betaine | 0.83783974 | 0.00035285 |
| N-carbamoyl-L-aspartate | -2.6848358 | 0.0003478 |
| L-Tyrosine | -0.6204377 | 0.00051624 |
| 2,6-Dimethylaniline | -0.5821477 | 0.00036365 |
| D-Erythrose 4-phosphate | -2.3074263 | 0.00167401 |
| Quinic acid | -0.6194126 | 0.00035109 |
| D-Glucuronic acid | -1.2632343 | 0.00047923 |
| D-Xylulose 5-phosphate | -3.9155882 | 0.00175392 |
| D-Glucose 6-phosphate | -1.5438449 | 0.00045262 |
| Hexanoyl Glycine | -1.4881461 | 0.00050459 |
| Adrenochrome | -1.9717676 | 0.0006879 |
| Pyridoxamine | 1.38422773 | 0.00116066 |
| trans-Cinnamic acid | -0.7764706 | 0.00050073 |
| Methionine sulfoxide | 0.70451897 | 0.00091479 |
| Purine | -0.5021682 | 0.00048785 |
| Tyramine | -0.7829354 | 0.00044941 |
| (S)-Leucic acid | 1.2850687 | 0.00092225 |
| Hypoxanthine | -0.8843095 | 0.00055204 |
| S-Adenosyl-L-homocysteine | -1.7142481 | 0.0019987 |
| Tauroursodeoxycholic acid | -3.4853649 | 0.00142019 |
| 3-Indoleacrylate | -1.1080519 | 0.00425003 |
| Cuminaldehyde | -0.5573184 | 0.00054815 |
| Porphobilinogen | -0.5239112 | 0.00103338 |
| 3-(2-Hydroxyphenyl)propionic acid | -0.6161652 | 0.00055761 |
| Xanthurenic Acid | -1.0005653 | 0.00393722 |
| 1-Methylguanine | 1.19205066 | 0.00054562 |
| L-Cysteine | -1.7534067 | 0.00062956 |
| 2-(Formylamino)Benzoic Acid | -0.5688703 | 0.00089344 |
| Taurocholic acid | -2.5240285 | 0.00082483 |
| Hydroxypyruvic acid | -0.8115991 | 0.00062168 |
| Phthalate | -0.7899827 | 0.00060942 |
| Pantetheine | -3.8218458 | 0.00065775 |
| Kynurenic acid | 2.0547139 | 0.00343998 |
| Urea | 0.51149691 | 0.00063226 |
| 7-Methylguanine | -0.715865 | 0.00091695 |
| N,N,N-trimethyllysine | 0.73470441 | 0.00393018 |
| Thiamine | -1.2753792 | 0.00105143 |
| L-Kynurenine | 1.68549765 | 0.00407013 |
| Orotic Acid | 1.77409917 | 0.0011857 |
| scyllo-Inositol | -0.4417671 | 0.00524898 |
| D-Arabinose 5-phosphate | -3.658604 | 0.00426126 |
| Guanine | -1.8017745 | 0.00274105 |
| Dipicolinic acid | -0.2793304 | 0.0009415 |
| Melamine | -1.7265912 | 0.00095809 |
| Ribose 1-phosphate | -3.6536511 | 0.00472361 |
| L-Cysteine-glutathione gisulfide | -0.578759 | 0.00409544 |
| Isobutyryl carnitine | 2.51297124 | 0.00707045 |
| Glycyl-L-valine | -1.6335988 | 0.00160667 |
| 2-Oxoadipic acid | -0.794431 | 0.00220971 |
| 3-Methylcrotonyl Glycine | -0.8885492 | 0.00132968 |
| Nelarabine | 1.20229477 | 0.00354513 |
| 6-Methylmercaptopurine | -0.5522065 | 0.0019862 |
| Phloretate | -0.5382343 | 0.00161229 |
| 2-Methylsuccinic Acid | -1.0689893 | 0.00123154 |
| 5-Hydroxyhexanoic Acid | 1.22015407 | 0.00225877 |
| 5-Methyluridine | -2.6753382 | 0.00538082 |
| Caffeylalcohol | -0.5798338 | 0.00226113 |
| CTP | -0.88708 | 0.00243516 |
| geranyl pp | 2.99936366 | 0.00154667 |
| L-allo-Isoleucine | -0.7370957 | 0.00192696 |
| Nicotinic Acid | -1.6161486 | 0.00429392 |
| Guanosine monophosphate | -2.7602056 | 0.00506048 |
| Enterodiol | 2.07975185 | 0.0015326 |
| Carnosine | -2.0664695 | 0.00227406 |
| Sebacic acid | -0.9985457 | 0.00471113 |
| alpha-D-Galactose 1-phosphate | -1.7222496 | 0.00223212 |
| D-Lactic acid | -0.9294523 | 0.00682628 |
| 2-Deoxy-D-galactose | -1.6344052 | 0.0022467 |
| 2-Phenylglycine | -1.5955494 | 0.0033341 |
| Gly-Phe | -2.3768463 | 0.00239726 |
| D-Proline | -0.4366445 | 0.00334327 |
| L-Leucine | -0.472588 | 0.00245372 |
| Oleoylcarnitine | 1.29381261 | 0.003347 |
| cis-Aconitic acid | -1.6055342 | 0.00796002 |
| N-Formylkynurenine | 1.82054032 | 0.00318143 |
| Creatine | -0.964878 | 0.00841392 |
| Maleic Acid | -0.5301127 | 0.00394001 |
| 2-Hydroxyisocaproic Acid | 1.21516124 | 0.00344897 |
| N-Methylnicotinamine | -0.797146 | 0.00524478 |
| Homoarginine | 1.03400091 | 0.00317087 |
| 2-Hydroxy-6-Aminopurine | -1.2872748 | 0.00496154 |
| L-Arginine | -1.6941785 | 0.00660117 |
| Phosphocreatine | 0.67843467 | 0.00676645 |
| Cytosine | 1.39294629 | 0.00492562 |
| Pyrimidine | -1.1028954 | 0.00434786 |
| Hexanoylcarnitine | -1.1839734 | 0.00318302 |
| UDP-galactose | -1.4397685 | 0.00315158 |
| Ribulose-5-phosphate | -3.8715007 | 0.01020156 |
| Nicotinate ribonucleoside | 1.38192189 | 0.0063057 |
| 5-Aminopentanoate | -0.8145966 | 0.00374848 |
| DL-o-Tyrosine | -0.6740906 | 0.00366762 |
| trans-3-Hydroxy-L-proline | -0.7231779 | 0.00522282 |
| Linoleoylcarnitine | 0.97788759 | 0.00453828 |
| L-Histidinol | 1.66980838 | 0.01104297 |
| N-Acetyl-L-glutamic acid | 0.63527559 | 0.01023747 |
| Tryptamine | 1.82245132 | 0.01235868 |
| 5-Aminosalicylate | -0.7656181 | 0.00386469 |
| 6-Sialyllactose | -1.5904245 | 0.0037951 |
| 3-Methyladipic acid | -1.4892722 | 0.0127813 |
| D-Fructose 1,6-bisphosphate | -1.0170812 | 0.01330287 |
| D-Ribose 5-phophate | -3.7377427 | 0.00956472 |
| Fexofenadine | -1.2682437 | 0.00467604 |
| L-Ornithine | -0.4445577 | 0.00459155 |
| 6-Aminohexanoate | -0.7022932 | 0.0048447 |
| 1-Methylhistamine | 1.27037869 | 0.01547205 |
| Val-Ser | -2.0285969 | 0.0067149 |
| isoleucine | -0.4312774 | 0.00486996 |
| 4-Acetamidobutanoate | -1.3387308 | 0.01430441 |
| Pyrrole-2-carboxylate | -0.8964421 | 0.01266551 |
| 10-Formyl-Thf | 1.16773783 | 0.01022655 |
| 8-Isoprostaglandin E1 | 1.02658806 | 0.00613761 |
| Isobutyrylglycine | -0.6420494 | 0.00639734 |
| 2-Hydroxy-2-methylbutanoic acid | -0.840202 | 0.01055186 |
| L-allo-Threonine | -0.3290456 | 0.00562552 |
| Pimelic acid | -1.476447 | 0.01722634 |
| 2-Phenylethylamine | 2.01375557 | 0.01667701 |
| 3'-Adenylic acid | -0.4411374 | 0.00635798 |
| 5-Aminolevulinate | -0.4647705 | 0.00639411 |
| Stachydrine | -0.5719772 | 0.00644845 |
| Protocatechualdehyde | -1.2688783 | 0.00631165 |
| 3-Hydroxy-butyryl carnitine | -0.9350186 | 0.01302046 |
| Cyprodinil | -0.3179689 | 0.00964393 |
| Pseudouridine | 0.99946834 | 0.01712967 |
| 15-Hete | 1.78545474 | 0.00771816 |
| 12(S)-HETE | 1.78669389 | 0.00751494 |
| N-Methyl-a-aminoisobutyric acid | -0.4069239 | 0.00773616 |
| Choline | -0.3852617 | 0.00903424 |
| 1-Aminocyclopropane-1-carboxylic acid | -0.5036778 | 0.00954079 |
| beta-Nicotinamide mononucleotide | -0.7800273 | 0.01665242 |
| L-Lactic acid | -0.8557469 | 0.01695878 |
| Ethylmalonate | 0.99251774 | 0.01928855 |
| Glycyl-L-leucine | -2.0683381 | 0.00921297 |
| trans-2-Butene-1,4-dicarboxylic Acid | -1.7411491 | 0.01653843 |
| Methylguanidine | -0.3729298 | 0.00839651 |
| Maltotriose | -2.257062 | 0.01463208 |
| N-Acetyl-L-methionine | -1.2000964 | 0.01966399 |
| Oxypurinol | -0.5462172 | 0.00936845 |
| N-Acetylneuraminic acid | -0.9899921 | 0.0235714 |
| L-SelenoMethionine | 0.05611396 | 0.01530658 |
| Alloxan | -0.7495915 | 0.02080095 |
| Spermidine | -0.6794961 | 0.01572792 |
| 4-Hydroxy-2-oxoglutaric acid | -0.6303607 | 0.02277304 |
| L-Histidine | -0.6085631 | 0.01226187 |
| dCMP | 1.46648723 | 0.01641678 |
| 2-Pyrrolidinone | -0.552243 | 0.01239523 |
| L-Tyrosinemethylester | -1.4205608 | 0.01196417 |
| Sedoheptulose 1,7-bisphosphate | -1.0330658 | 0.01213135 |
| Methionine | -0.4061739 | 0.01257898 |
| 5-Methylcytosine | 0.51857047 | 0.02787522 |
| Glycine | -0.4078734 | 0.02388652 |
| Acetyl phosphate | -2.4408379 | 0.0345929 |
| N-Acetyl-D-glucosamine | -0.8417758 | 0.01307874 |
| Adenine | -0.564069 | 0.01792571 |
| 2-Hydroxy-3-methylbutanoic acid | -0.8276528 | 0.02871035 |
| Galactinol | -2.0612888 | 0.0336042 |
| 1,4-Dihydro-1-Methyl-4-Oxo-3-Pyridinecarboxamide | -0.4944631 | 0.01755214 |
| 3-Ureidopropionate | -0.4063163 | 0.01754263 |
| Urocanic acid | -0.926095 | 0.02436144 |
| Dimethyl fumarate | -1.6989813 | 0.01719456 |
| Ribitol | -0.5696797 | 0.03145144 |
| Phenylacetylglycine | 0.9187725 | 0.01803766 |
| L-Lysine | 0.47750236 | 0.02477523 |
| Cadaverine | 0.58122052 | 0.03755883 |
| Adenosine | -0.6046071 | 0.02513048 |
| 13-HPODE | 0.82395143 | 0.04073134 |
| Stearoylcarnitine | 1.55349451 | 0.03636437 |
| Vidarabine | -0.6326166 | 0.02327909 |
| Spermine | 0.97635787 | 0.02069069 |
| 3-Phosphoglyceric acid | -2.3270135 | 0.04669022 |
| L-Rhamnose monohydrate | -1.101318 | 0.03550189 |
| N alpha-Acetyl-L-Arginine | 1.29237526 | 0.04500351 |
| 9(S)-HPODE | -1.3620089 | 0.03529799 |
| Xanthine | -0.2749516 | 0.02232074 |
| UDP-N-acetylglucosamine | -1.214136 | 0.02335469 |
| Imidazole | -0.5298528 | 0.02891296 |
| Glycocholic acid | -2.6029092 | 0.02868232 |
| UDP-D-glucose | -0.8724742 | 0.02386985 |
| Thymidine 5'-diphosphate | -1.1531117 | 0.02811433 |
| 1,3-Diaminopropane | -0.6716807 | 0.03939709 |
| Creatinine | -0.88555 | 0.04044195 |
| L-Cystathionine | -0.9917176 | 0.04461413 |
| Adenylocuccinic Acid | -1.9105716 | 0.04130661 |
| Leu-Pro | -1.8043387 | 0.04705402 |
| Glycodeoxycholic acid | -1.8082525 | 0.04910291 |

**Table S2. Differentially metabolites in serum samples between ConA and NC mice.**

| Name | log2FC | P value |
| --- | --- | --- |
| Xanthine | -11.61283 | 1.73E-12 |
| L-Canavanine | -4.2991301 | 2.55E-07 |
| (S)-3-Aminoisobutyrate | 3.39968537 | 3.02E-08 |
| 3-Oxo-7alpha,12alpha-hydroxy-5beta-cholanoic acid | -3.8574705 | 1.55E-05 |
| N-Formyl-L-methionine | -3.6525235 | 9.54E-06 |
| Indole-3-acetamide | -3.555434 | 8.04E-10 |
| Prostaglandin E1 | -3.1480122 | 2.51E-09 |
| 7-Ketocholesterol | -2.8689032 | 1.47E-07 |
| 2,3-Dihydroxybenzoic acid | -2.7223149 | 1.87E-05 |
| 1,4-Dihydro-1-Methyl-4-Oxo-3-Pyridinecarboxamide | -2.4135775 | 0.00213594 |
| Inosine | -2.2832523 | 0.02271518 |
| Gly-Phe | -2.2352406 | 0.0001142 |
| N-Formylkynurenine | 3.31235494 | 1.58E-08 |
| Hydroquinone | -2.0693268 | 1.28E-05 |
| Indolin-2-one | -2.0525087 | 3.04E-06 |
| 4-Pyridoxic acid | 3.01376233 | 8.03E-12 |
| Adenosine | 2.92725717 | 1.04E-08 |
| Hyodeoxycholate | -1.8419172 | 0.00118183 |
| Riboflavin-5-phosphate | 2.87925255 | 2.28E-07 |
| 1-Methylnicotinamide | -1.8371081 | 0.03467733 |
| Indoxylsulfuric acid | -1.8318772 | 5.21E-06 |
| D-Xylulose 5-phosphate | -1.830592 | 1.70E-06 |
| Vidarabine | 2.87156755 | 2.98E-09 |
| S-Sulfo-L-cysteine | 2.81666696 | 3.40E-09 |
| Ribose 1-phosphate | -1.8046964 | 3.11E-06 |
| Hydroxypyruvic acid | -1.7865615 | 4.55E-07 |
| 5-Methoxytryptophol | 2.78969606 | 3.65E-09 |
| 13-HPODE | 2.74847537 | 0.01913732 |
| 2-Phenylethylamine | 2.5752516 | 0.00016603 |
| 9(S)-HPODE | 2.54957194 | 1.86E-09 |
| Taurocholic acid | 2.50122055 | 4.04E-05 |
| 3-Methylcrotonyl Glycine | -1.7791482 | 1.24E-07 |
| Guanidinoacetic acid | -1.7520829 | 1.21E-09 |
| N-Carbamyl-L-glutamicacid | -1.7284489 | 1.51E-08 |
| Taurochenodeoxycholic acid | 2.47008081 | 6.32E-07 |
| Homogentisic Acid | 2.42106354 | 0.00012682 |
| D-Glucose 6-phosphate | 2.22305528 | 1.61E-05 |
| Pyridoxal 5-phosphate | 2.15945095 | 1.17E-06 |
| Tauroursodeoxycholic acid | 2.12072293 | 4.14E-06 |
| D-Arabinose 5-phosphate | -1.6700935 | 5.57E-06 |
| N-Acetylvaline | -1.6263924 | 6.35E-05 |
| 21-Deoxycortisol | -1.6107372 | 1.80E-07 |
| Hexanoyl Glycine | -1.6021741 | 2.00E-10 |
| 5-Hydroxyindoleacetate | -1.5910583 | 0.00010621 |
| Taurohyocholic acid | 2.11881118 | 1.03E-05 |
| Xanthosine | -1.5887404 | 0.00093731 |
| Hypoxanthine | -1.5831607 | 0.02626431 |
| Tauro-alpha-Muricholic acid | 2.05678235 | 1.21E-05 |
| D-Ribose 5-phophate | -1.5776717 | 7.61E-06 |
| Guanosine monophosphate | 2.00436355 | 7.79E-05 |
| L-Cysteine-glutathione gisulfide | 1.82493032 | 6.74E-05 |
| Oxypurinol | -1.561906 | 0.0293686 |
| Ursodeoxycholic acid | -1.5595632 | 0.00322186 |
| 2-Methylpentanedioic acid | -1.548239 | 0.02554491 |
| Cytarabine | -1.5363068 | 4.42E-09 |
| L-Ascorbate | -1.5303494 | 0.00047507 |
| dAMP | 1.78135497 | 0.00266901 |
| cholesteryl sulfate | 1.69848329 | 0.00105161 |
| Cytidine | -1.5288886 | 3.02E-07 |
| dCMP | 1.54836254 | 3.39E-05 |
| Ribulose-5-phosphate | -1.5004682 | 2.29E-05 |
| Inosine 5'-Monophosphate | -1.4834731 | 6.21E-08 |
| Tricarballylic acid | -1.4184849 | 2.92E-05 |
| p-Hydroxybenzaldehyde | 1.49112023 | 3.77E-05 |
| Spingosine-1-phoshate | -1.4048748 | 5.01E-07 |
| 3-Hydroxy-butyryl carnitine | -1.341182 | 7.52E-09 |
| 12(S)-HETE | -1.3086962 | 6.80E-07 |
| 15-Hete | -1.2945251 | 7.87E-07 |
| 3-O-Feruloylquinic acid | 1.41043959 | 9.20E-07 |
| alpha-D-Galactose 1-phosphate | 1.40733036 | 0.00030118 |
| Ergothioneine | 1.29125151 | 0.00239605 |
| Enterodiol | -1.2711347 | 1.53E-06 |
| Urocanic acid | -1.2548062 | 6.50E-05 |
| 5'-Deoxy-5'-(methylthio)adenosine | -1.2191225 | 5.54E-11 |
| Taurolithocholic acid | -1.1138724 | 1.76E-06 |
| Phosphopyruvic acid | 1.2772605 | 8.00E-05 |
| Lysopc 17:0 | -1.0685459 | 2.49E-09 |
| gamma-Butyrolactone | -1.0684914 | 0.00023035 |
| Imatinib | -1.0363899 | 9.40E-09 |
| N-Acetyl-L-tyrosine | 1.27139708 | 1.39E-06 |
| 3-Ureidopropionate | 1.18488241 | 3.64E-07 |
| Lysopc 16:1 | -1.0298333 | 7.32E-09 |
| 9,10-Dihome | 1.1603943 | 3.70E-06 |
| Orotidine | -1.0028447 | 5.22E-08 |
| Nicotinate ribonucleoside | -0.9705775 | 0.00206377 |
| Glycerol 1-hexadecanoate | -0.9663623 | 6.60E-05 |
| Lysopc 15:0 | -0.948486 | 1.11E-09 |
| Myristic acid | 1.03124187 | 0.0082542 |
| All-cis-4,7,10,13,16-docosapentaenoic acid | -0.9435226 | 0.00029205 |
| 3'-Adenylic acid | 1.00965071 | 1.99E-08 |
| 3-Ketodihydrosphingosine | 1.00441476 | 0.00089728 |
| Decanoic acid | -0.9158676 | 1.45E-05 |
| Stearoylcarnitine | 0.97676037 | 8.40E-08 |
| Isonicotinic acid | 0.93996729 | 1.98E-07 |
| Pantothenic acid | 0.93987469 | 1.73E-06 |
| Metformin | -0.9001521 | 0.0045742 |
| Isobutyryl carnitine | 0.91617515 | 2.91E-05 |
| 3-PRENYL-4-HYDROXYACETOPHENONE | -0.8942044 | 8.71E-05 |
| Hydantoin-5-propionic acid | -0.8657349 | 1.61E-05 |
| 5-Oxo-ETE | -0.8638128 | 6.70E-05 |
| Lysopc 20:0 | -0.8395023 | 3.47E-06 |
| 2-Acetylphloroglucinol | -0.8233492 | 0.00668805 |
| D-3-Phenyllactic acid | -0.8101752 | 7.92E-05 |
| glutathione disulfide | 0.90512003 | 0.00122564 |
| dUMP | 0.86913305 | 0.00341445 |
| Sarcosine | 0.85475035 | 3.39E-06 |
| alpha-Ketoglutaric acid | 0.8157167 | 0.00033313 |
| Nicotinamide | 0.80082922 | 1.95E-06 |
| Lysopa 16:0 | -0.7953659 | 3.00E-07 |
| gamma-Tocopherol | -0.7864076 | 0.0003192 |
| O-Desmethylnaproxen | 0.78775242 | 1.78E-06 |
| Lysopc 14:0 | -0.7793309 | 6.72E-10 |
| L-Pyroglutamic acid | 0.76072177 | 2.02E-09 |
| N,N,N-trimethyllysine | 0.75392322 | 7.38E-08 |
| Phosphocholine | -0.7732792 | 4.15E-05 |
| 4-Oxoretinol | -0.7718629 | 0.00029899 |
| Asp-Phe methyl ester | 0.74325308 | 3.75E-08 |
| LysoPC 20:2 | -0.7387806 | 4.48E-06 |
| 2-Hydroxy-2-methylbutanedioic acid | -0.7123352 | 0.00159882 |
| N-Acetyl-aspartic acid | -0.7035137 | 2.32E-06 |
| Trigonelline | -0.7017079 | 0.00130366 |
| 9-KODE | -0.6901063 | 0.00050478 |
| Arachidonoylcarnitine | 0.74217883 | 4.22E-05 |
| Glucotropaeolin | 0.72760868 | 0.00139528 |
| Isovalerylcarnitine | 0.70949181 | 0.00010811 |
| D-Glucosamine 6-phosphate | 0.69472613 | 0.00024939 |
| Allantoin | 0.68712191 | 5.56E-07 |
| Deoxycorticosterone | -0.671794 | 0.00203989 |
| L-Thyroxine | -0.6711012 | 0.00015392 |
| N-Acetyl-L-glutamic acid | 0.67488123 | 8.03E-08 |
| Sebacic acid | -0.6646594 | 1.10E-06 |
| Pyrophosphate | 0.67100482 | 0.00074985 |
| Porphobilinogen | -0.6612482 | 0.00049987 |
| 3-Methylindole | -0.6579135 | 0.00049612 |
| N-acetyl-glutamate | 0.65523255 | 4.68E-07 |
| cis-7-Hexadecenoic Acid | -0.6456586 | 0.00232561 |
| dTMP | -0.6156773 | 0.00865957 |
| L-Histidine | -0.5977498 | 0.00128867 |
| Choline | -0.5892822 | 1.36E-05 |
| Hydroxyphenyllactic acid | -0.5881401 | 0.00262709 |
| 2-(Dimethylamino)Guanosine | 0.63920542 | 7.86E-07 |
| L-Tryptophan | -0.5842113 | 0.00068258 |
| alpha-Ketocaproic acid | 0.62923503 | 0.00052016 |
| Succinic anhydride | 0.59999334 | 2.65E-07 |
| Palmitoylethanolamide | 0.55069952 | 4.59E-06 |
| Acetylcholine | 0.5483795 | 0.00057015 |
| Tiglic acid | 0.51659597 | 3.53E-05 |
| 1-Oleoyl-Sn-Glycero-3-Phosphocholine | -0.5668972 | 7.68E-07 |
| Erythronolactone | 0.50639503 | 0.00594334 |
| Lysope 16:0 | -0.5624464 | 1.87E-06 |
| 2,6-Dimethylaniline | 0.4985036 | 3.05E-06 |
| 3-(2-Hydroxyphenyl)propionic acid | 0.49724697 | 2.07E-07 |
| 2-(Formylamino)Benzoic Acid | 0.49245999 | 3.29E-07 |
| N-Acetylmannosamine | -0.5602512 | 0.00415893 |
| 5-Methoxyindole-3-acetic acid | -0.5556536 | 0.00098401 |
| Phloretate | 0.49120609 | 6.86E-08 |
| Isoquinoline | -0.5516617 | 0.00092009 |
| Lysopc 20:4 | -0.5451342 | 4.96E-08 |
| Itaconic acid | -0.5434317 | 0.00027891 |
| D-threo-Isocitric acid | -0.5430322 | 5.35E-05 |
| Pimelic acid | -0.5370226 | 0.00028716 |
| 3-Methyladipic acid | -0.5364888 | 0.00022149 |
| 3-Hydroxyisovalerate | 0.48834174 | 0.00810202 |
| 7-Methylguanine | 0.4840612 | 8.37E-08 |
| 5-Aminosalicylate | -0.5363157 | 0.00555771 |
| 2-Aminoethylphosphonate | -0.524325 | 0.00511729 |
| 20-Carboxy-Leukotriene B4 | -0.5226915 | 0.00273118 |
| 3-Indoleacrylate | -0.5213748 | 0.00087372 |
| Methionine | -0.4905687 | 0.00269293 |
| Phthalate | 0.47490084 | 3.56E-07 |
| N,N-Dimethylglycine | -0.4755063 | 5.03E-07 |
| Xanthurenic Acid | -0.469123 | 0.00274575 |
| N-Acetylthreonine | -0.46783 | 0.00079556 |
| 1-Palmitoyl-Sn-Glycero-3-Phosphocholine | -0.4670372 | 6.59E-09 |
| 6-Methylmercaptopurine | 0.47211265 | 1.25E-08 |
| Taurine | -0.4624747 | 0.00029467 |
| N-(5-Aminopentyl)acetamide | -0.458039 | 0.00434178 |
| Furfural | -0.4525122 | 0.00063362 |
| L-Glutamate | 0.45824885 | 0.00385917 |
| 3-Methyl-2-oxobutanoic acid | 0.45192346 | 0.00034032 |
| Hydroxyglutaric acid | -0.4350038 | 0.00012912 |
| 4-Amino-5-imidazolecarboxamide | -0.4332279 | 0.00167842 |
| trans-Cinnamic acid | 0.43731753 | 1.81E-06 |
| N4-Acetylcytidine | 0.418096 | 0.00889465 |
| Betaine | -0.4173764 | 0.00509412 |
| Purine | 0.41659349 | 2.44E-07 |
| trans-Elaidic acid | 0.41606379 | 0.00131511 |
| Cuminaldehyde | 0.40907104 | 3.18E-06 |
| 11-Ketoetiocholanolone | -0.4149457 | 0.0008598 |
| 1-Methylhistidine | 0.39179662 | 0.00435856 |
| Lysopc 18:2 | -0.4071724 | 1.77E-09 |
| Lysopc 18:3 | -0.4062796 | 2.05E-11 |
| 3-Aminoisobutyric acid | -0.3750448 | 0.00163233 |
| L-Threonine | 0.3852521 | 0.00404899 |
| Inositol | -0.3736671 | 0.00558033 |
| Aminomalonic acid | 0.36649094 | 0.00229458 |
| L-Lysine | 0.36609671 | 0.00025243 |
| Ala-gly | 0.30958077 | 0.00026369 |
| 1-Stearoyl-Sn-Glycerol-3-Phosphocholine | -0.342311 | 1.49E-05 |
| Fexofenadine | -0.3265387 | 0.00065805 |
| L-Valine | 0.28537511 | 0.00561674 |
| Thymidine 3,5-cyclic monophosphate | 0.27339531 | 0.00297268 |
| Octanedioic acid | -0.3255333 | 0.00377266 |
| DL-3,4-Dihydroxyphenyl glycol | -0.3227765 | 0.00111547 |
| Cycloleucine | 0.24488196 | 0.00112336 |
| Nelarabine | 0.22382762 | 0.00279808 |
| L-Fucose | -0.3122914 | 0.00641722 |
| N-Acetylglycine | -0.3063237 | 0.00386323 |
| isoleucine | -0.2810191 | 0.00595214 |
| 11-cis-Retinol | -0.247265 | 3.14E-05 |
| Adipic Acid | 0.17891809 | 0.00570541 |
| Isoproterenol | -0.2196587 | 0.00162856 |
| Cholesterol | -0.1767933 | 0.0021624 |

**T****able S3. Differentially metabolites in serum samples between the AIH and HC groups.**

| Name | log2FC | P value |
| --- | --- | --- |
| Taurocholic acid | -6.6451065 | 1.93E-18 |
| 1-Methylxanthine | 2.75716199 | 5.97E-05 |
| Glycoursodeoxycholic acid | -4.307775 | 3.29E-18 |
| Glycochenodeoxycholic acid | -4.3054698 | 2.31E-18 |
| 5'-Deoxy-5'-(methylthio)adenosine | -3.9381306 | 0.00617694 |
| S-Methyl-5'-thioadenosine | -3.8907996 | 0.00587561 |
| Glycodeoxycholic acid | -3.3711285 | 1.62E-05 |
| Glutathione | 2.38939185 | 5.49E-23 |
| Quinone | -3.0355658 | 4.82E-21 |
| Cholic acid | -2.8346637 | 0.00028767 |
| D-Glucuronic acid | -2.7405398 | 8.53E-10 |
| Inosine | -2.7232096 | 0.00017638 |
| Hexadecanedioate | -2.6443526 | 9.07E-17 |
| N-acetyl-glutamate | 1.89457482 | 2.37E-19 |
| Glycolithocholic acid | -2.329058 | 9.59E-06 |
| Allopurinol | -2.3063189 | 7.92E-06 |
| Methyl Benzoate | -2.2308531 | 2.63E-13 |
| Estrone | -2.032519 | 6.86E-05 |
| Tetrahydrocorticosterone | -1.9436305 | 0.01480778 |
| 1,4-Dihydro-1-Methyl-4-Oxo-3-Pyridinecarboxamide | -1.8992376 | 3.25E-08 |
| Oxypurinol | -1.8956567 | 3.79E-07 |
| Xanthine | -1.8742573 | 5.99E-08 |
| CMPF | 1.53271845 | 4.66E-09 |
| Guanosine monophosphate | -1.7474713 | 2.64E-10 |
| L-Cystathionine | -1.7123911 | 7.33E-12 |
| Tetradecanedioic acid | -1.6661986 | 9.91E-13 |
| Arachidic acid | -1.6063237 | 5.09E-13 |
| Homogentisic Acid | -1.5885043 | 1.68E-10 |
| L-Canavanine | -1.5062607 | 0.02443507 |
| Hypoxanthine | -1.3540794 | 0.00017743 |
| Methionine sulfoxide | -1.3349361 | 1.22E-15 |
| Hydroxypyruvic acid | -1.2540373 | 0.00814345 |
| Palmitoleic acid | -1.246129 | 0.00067528 |
| 5'-Adenylic acid | -1.2207172 | 2.73E-07 |
| L-Theanine | 1.35782665 | 0.0001944 |
| Phe-Phe | 1.24507101 | 1.30E-15 |
| 7-Ketocholesterol | 1.15648302 | 1.07E-07 |
| Nelarabine | -1.1632773 | 2.86E-13 |
| N-Formylkynurenine | -1.1582901 | 1.94E-15 |
| L-Cysteine | 1.09871556 | 2.46E-10 |
| Vitamin B2 | -1.155775 | 1.85E-13 |
| Adenosine 5'-Diphosphate | -1.1399388 | 0.00278326 |
| Hydrocortisone acetate | 1.01558672 | 9.00E-12 |
| Anthranilic acid | -1.1336195 | 0.00015268 |
| Fexofenadine | 0.94807673 | 3.31E-15 |
| (3-Methoxy-4-hydroxyphenyl)ethylene glycol sulfate | -1.1185092 | 1.16E-11 |
| L-Saccharopine | 0.92658853 | 2.81E-15 |
| dGDP | -1.1176968 | 0.00336638 |
| 1-Methylguanosine | -1.1117528 | 4.77E-09 |
| 2,6-Dihydroxybenzoic acid | -1.0957345 | 0.00180842 |
| 4-Hydroxyphenyllactate | -1.0885449 | 1.44E-11 |
| Hydroxyphenyllactic acid | -1.0768715 | 1.20E-11 |
| APS | -1.0652376 | 0.00627734 |
| 5alpha-Pregnane-3,20-dione | -1.0273561 | 2.43E-07 |
| Tartaric Acid | -1.0191635 | 0.0231754 |
| 11-Ketoetiocholanolone | -1.0102943 | 2.25E-08 |
| Guggulsterone | 0.92330168 | 6.89E-18 |
| S-Adenosyl-L-homocysteine | -1.0096038 | 3.31E-05 |
| 11beta-Hydroxytestosterone | -0.9970563 | 1.16E-14 |
| 1,4-Naphthoquinone | 0.91846695 | 1.70E-11 |
| Ergothioneine | 0.85712763 | 3.19E-08 |
| 1-Methylguanine | -0.99084 | 2.12E-10 |
| Estriol | -0.9908018 | 2.27E-15 |
| D-3-Phenyllactic acid | -0.9874461 | 1.02E-05 |
| alpha-Ketocaproic acid | 0.78838721 | 3.49E-15 |
| L-Aspartic acid | 0.77989426 | 9.53E-11 |
| Asaraldehyde | -0.9737414 | 1.37E-06 |
| L-Erythrulose | -0.9678043 | 4.97E-06 |
| Phosphocreatine | 0.77302878 | 1.97E-08 |
| 2-Methylpentanedioic acid | -0.9342161 | 0.00052006 |
| 5-Aminosalicylate | -0.8893045 | 1.29E-05 |
| 1-Palmitoyl-Sn-Glycero-3-Phosphocholine | 0.67206713 | 5.32E-12 |
| 5beta-Androstan-17beta-ol-3-one | -0.8739878 | 5.86E-14 |
| cis-Aconitic acid | -0.869382 | 6.04E-11 |
| N4-Acetylcytidine | -0.8638985 | 2.71E-08 |
| Cortisone | 0.63843874 | 1.21E-05 |
| Deoxycytidine | -0.8457848 | 1.25E-13 |
| 2-Hydroxy-3-methylbutanoic acid | -0.8443854 | 3.53E-06 |
| 1-Oleoyl-Sn-Glycero-3-Phosphocholine | 0.61008024 | 6.77E-10 |
| alpha-D-Galactose 1-phosphate | 0.58332216 | 1.83E-06 |
| Isophorone | -0.8439056 | 1.55E-08 |
| DL-o-Tyrosine | -0.8407447 | 1.25E-14 |
| 3-Methyl-2-oxobutanoic acid | 0.58295784 | 3.18E-14 |
| 2-Hydroxycinnamate | -0.8299553 | 1.19E-15 |
| L-Arginine | -0.8297348 | 2.05E-06 |
| Cystine | -0.8265578 | 1.20E-11 |
| L-Octanoylcarnitine | 0.5699668 | 0.00056086 |
| Tyramine | -0.8260361 | 6.07E-07 |
| Indole-3-acetamide | -0.8233364 | 2.33E-06 |
| Imidazoleacetic acid | -0.7955076 | 1.91E-12 |
| 1-(4-Methoxyphenyl)-2-propanone | -0.7804564 | 1.81E-14 |
| 5-Hydroxyindoleacetate | -0.7601062 | 2.98E-05 |
| N-Acetyl-L-tyrosine | -0.7389285 | 5.00E-06 |
| 11-cis-Retinol | 0.54966168 | 1.87E-07 |
| N-Acetylglycine | -0.7212102 | 1.03E-07 |
| Homocysteic acid | -0.7211253 | 8.46E-13 |
| Cytidine | 0.51291835 | 2.17E-05 |
| Orotic Acid | -0.7007515 | 1.81E-06 |
| Decanoylcarnitine | 0.47144919 | 0.00272317 |
| Imatinib | 0.44760429 | 4.77E-06 |
| D-Glucose 6-phosphate | -0.6704878 | 1.52E-07 |
| Picolinic acid | -0.6533308 | 6.24E-05 |
| 5-Methoxyindole-3-acetic acid | 0.42623245 | 6.76E-08 |
| 6-Phospho-D-glucono-1,5-lactone | 0.42144861 | 4.48E-05 |
| 5-Hydroxylysine | -0.6532057 | 2.57E-08 |
| D-Glucosamine | 0.40449669 | 1.43E-06 |
| Orotidine | -0.643535 | 2.26E-05 |
| Xanthurenic Acid | 0.38461288 | 4.92E-07 |
| Porphobilinogen | 0.38245934 | 1.31E-07 |
| Citric acid | -0.6421745 | 5.63E-07 |
| 2-Phenylbutyric acid | -0.6348714 | 1.96E-07 |
| Pantothenic acid | -0.6308791 | 1.16E-06 |
| Etiocholanolone | -0.6246056 | 1.02E-10 |
| Aldosterone | -0.6219509 | 7.59E-08 |
| Uridine | 0.37595737 | 8.11E-06 |
| D-Fructose 6-phosphate | -0.6043003 | 2.50E-08 |
| Stearic acid | 0.37444503 | 1.16E-05 |
| D-Mannose 6-phosphate | -0.6039047 | 6.03E-07 |
| D-threo-Isocitric acid | -0.5790279 | 6.90E-07 |
| L-Glutamate | -0.552275 | 1.63E-05 |
| Isocitrate | -0.551321 | 2.40E-06 |
| 2-Oxo-3-methyl-butyrate | -0.5498464 | 2.03E-06 |
| Quinic acid | -0.5393934 | 6.49E-07 |
| 2-Hydroxy-2-methylbutanedioic acid | -0.5213514 | 5.41E-06 |
| Pipecolic acid | -0.4880777 | 6.62E-05 |
| Triclosan | -0.4641962 | 4.22E-07 |
| 3-Methylindole | 0.35609039 | 1.16E-06 |
| S-Sulfo-L-cysteine | 0.34941674 | 2.94E-05 |
| N-Methyl-a-aminoisobutyric acid | 0.31625629 | 7.40E-08 |
| Hydrocortisone | 0.31340192 | 0.00128093 |
| Isoquinoline | 0.30698327 | 7.98E-06 |
| Adenosine | -0.4631947 | 6.57E-05 |
| DL-Norvaline | 0.30672877 | 1.43E-07 |
| L-Carnitine | 0.30527706 | 6.99E-05 |
| trans-Cinnamic acid | -0.4628738 | 2.06E-10 |
| Citraconic acid | -0.4594718 | 1.30E-06 |
| O-Acetyl-L-homoserine | 0.30378554 | 6.83E-05 |
| Vidarabine | -0.458648 | 5.72E-05 |
| L-Valine | 0.27970484 | 9.70E-07 |
| L-Normetanephrine | -0.4556416 | 8.51E-11 |
| Thymine | -0.4432712 | 0.00070686 |
| Cuminaldehyde | -0.4351275 | 2.60E-09 |
| Traumatic acid | -0.4169233 | 1.14E-06 |
| L-allo-Isoleucine | 0.25803123 | 2.08E-05 |
| Testosterone | -0.3952026 | 4.58E-05 |
| Glycerol-3-phosphate | 0.24261226 | 1.58E-05 |
| 2,6-Dimethylaniline | -0.3950814 | 5.16E-06 |
| Mefenamic acid | 0.17992179 | 3.14E-11 |
| Prostaglandin B2 | 0.1179677 | 3.88E-06 |
| Palmitaldehyde | -0.3856206 | 3.17E-19 |
| Metformin | -0.3807989 | 1.61E-09 |
| cis-gondoic acid | -0.3684982 | 0.00016681 |
| L-Citrulline | -0.3651552 | 1.77E-05 |
| 6-Methylmercaptopurine | -0.3614821 | 7.70E-08 |
| 2-(Formylamino)Benzoic Acid | -0.3516208 | 1.59E-07 |
| 7-Methylguanine | -0.3467082 | 3.49E-07 |
| Phthalate | -0.3465583 | 2.74E-07 |
| Phloretate | -0.3430837 | 2.97E-07 |
| D-Phenylalanine | -0.339263 | 7.01E-08 |
| D-Galactosamine | -0.335852 | 3.72E-09 |
| 3'-Adenylic acid | -0.3315794 | 1.34E-05 |
| Purine | -0.3273657 | 2.81E-06 |
| 4-Methylpentanoate | -0.2445919 | 0.00053459 |
| 3-Methylsalicylate | -0.2155915 | 4.87E-05 |
| 4-Hydroxyphenylacetate | -0.2055893 | 4.69E-05 |

**Table S6. Differentially metabolites in liver samples between the ConA+M and ConA groups.**

| Name | log2FC | P value |
| --- | --- | --- |
| N-Methyl-L-arginine | 0.38007867 | 0.00955659 |
| N-Methyl-a-aminoisobutyric acid | 0.25076881 | 0.04549083 |
| N-Acetylthreonine | 0.28397095 | 0.00451039 |
| N-Acetylglycine | 0.50764268 | 0.03981264 |
| Lysopc 18:2 | 0.31707685 | 0.02584095 |
| Lysopc 16:1 | 0.52392503 | 0.03104191 |
| Lysopc 15:0 | 0.52968953 | 0.01203077 |
| L-Alanyl-L-leucine | 1.2204397 | 0.04672637 |
| Isomaltose | 0.72134688 | 0.0266279 |
| Imidazoleacetic acid | 0.40459511 | 0.01530361 |
| Imatinib | 0.50564221 | 0.02470975 |
| Flavin Adenine Dinucleotide | 0.29032403 | 0.00923402 |
| Palmitoylethanolamide | -0.5481566 | 0.04786814 |
| 3-Hydroxy-DL-kynurenine | -0.1698787 | 0.04112362 |
| Dimethyl fumarate | 0.38560977 | 0.02211184 |
| Kynurenic acid | -1.2127474 | 0.03240273 |
| 7-Methylguanine | 0.27430612 | 0.01595412 |
| 5-Methoxyindole-3-acetic acid | 0.49710204 | 0.03221899 |
| 5-Hydroxylysine | 0.27931527 | 0.02252232 |
| 3-Nitro-L-Tyrosine | -0.1369032 | 0.01830265 |
| 2-(Formylamino)Benzoic Acid | 0.33134462 | 0.01730174 |
| 17alpha-Ethinyl estradiol | -0.7053692 | 0.04629063 |
| 16alpha-Hydroxytestosterone | -0.3075692 | 0.02760325 |
| 11beta-Hydroxytestosterone | 0.47741194 | 0.04271639 |
| 1-(4-Methoxyphenyl)-2-propanone | 0.48601463 | 0.04011301 |
| L-Normetanephrine | 0.33764392 | 0.01730002 |
| thiamine phosphate | -0.3927521 | 0.02321727 |
| 5-Methyluridine | 0.68932316 | 0.03945519 |
| Phloretate | 0.34583527 | 0.01697222 |
| 3-Methylindole | 0.41636604 | 0.03417826 |
| 11-Ketoetiocholanolone | -0.3516852 | 0.01194486 |
| 4-Acetamidobutanoate | 1.76752374 | 0.04773265 |
| trans-3-Hydroxy-L-proline | 0.38158781 | 0.02847867 |
| O-Acetyl-L-homoserine | 0.2077779 | 0.03713233 |
| Acetylcholine | 0.41027472 | 0.01930215 |
| Boldenone | -1.4283708 | 0.04354679 |
| Cortisone | 0.43240199 | 0.02140635 |
| L-Leucine | 0.23170547 | 0.03304083 |
| 3-Amino-4-methylpentanoic acid | -1.1152605 | 0.02917941 |
| Trimethylamine N-oxide | -1.0529024 | 0.0225794 |
| 1-Aminocyclopropane-1-carboxylic acid | 0.28954067 | 0.03774463 |
| Guanosine monophosphate | 0.72489936 | 0.03334439 |
| N-METHYL (-)EPHEDRINE | -0.5145429 | 0.03034201 |
| Glu-Glu | 0.55309185 | 0.03076812 |
| Cyprodinil | -0.330326 | 0.00084568 |
| Noradrenaline | -0.2579512 | 0.01394512 |
| Choline Glycerophosphate | 0.73486959 | 0.02548144 |
| gamma-Butyrolactone | 0.74988778 | 0.02701872 |
| Delta-Tridecalactone | -0.4051636 | 0.00696174 |
| Phthalate | 0.38091634 | 0.02173066 |
| Enterodiol | -1.4149481 | 0.0065316 |
| Maleic Acid | 0.38481505 | 0.03007766 |
| 11-cis-Retinol | 0.90502172 | 0.01629252 |
| Cocamidopropyl Betaine | -0.5784249 | 0.00220496 |
| 6-Methylmercaptopurine | 0.3444471 | 0.02678561 |
| L-Aspartic acid | 0.32085163 | 0.00715283 |
| Stearoylcarnitine | -1.0246452 | 0.00255082 |
| Isovalerylcarnitine | -0.5344538 | 0.02547783 |
| 4-Hydroxybenzoic acid | -0.2292624 | 0.00570488 |
| Xanthurenic Acid | 0.49388716 | 0.01856907 |
| alpha-D-Glucose | 0.64269862 | 0.02459814 |
| D-Glucuronic acid | 0.92722206 | 0.02533423 |
| Trehalose | -0.3950515 | 0.04963498 |
| UMP | 0.3085708 | 0.01563374 |
| Gluconolactone | 0.37999184 | 0.00995363 |
| N6-Acetyl-L-lysine | 0.46329027 | 0.04429362 |
| 12(S)-HETE | -1.2661225 | 0.0125911 |
| 8(R)-Hydroxy-(5Z,9E,11Z,14Z)-eicosatetraenoic acid | -1.3129498 | 0.00533975 |
| cis-4-Hydroxy-D-proline | 0.46537598 | 0.0163331 |
| Cytidine 3'-phosphate | -0.9635887 | 0.03991985 |
| D-Galactonic acid | 0.31146638 | 0.0421392 |
| D-Gluconic acid | 0.27284508 | 0.03295708 |
| L-Glutamate | 0.15472927 | 0.00319353 |
| DL-Norvaline | 0.26249044 | 0.02724512 |
| Riboflavin-5-phosphate | 0.64162185 | 0.00910721 |
| Sedoheptulose 1,7-bisphosphate | 0.29668359 | 0.00508772 |
| Thymidine 5'-diphosphate | 0.58872673 | 0.044494 |
| L-Thyroxine | -0.692257 | 0.02659306 |
| geranyl pp | 0.35441408 | 0.01349556 |
| Stearic acid | 0.43992008 | 0.03332598 |
| Monobutyl phthalate | -0.2595191 | 0.02130218 |
| Undecanedioic acid | -0.1667126 | 0.00733963 |
| Glycylglycine | 0.58416921 | 0.02696128 |
| L-Arabinose | 0.47418315 | 0.01656487 |
| DL-Serine | 0.25793125 | 0.01093363 |
| Hyodeoxycholate | -0.8496985 | 0.04301519 |
| Azelaic acid | -0.2621363 | 0.00188897 |
| Ursodeoxycholic acid | -0.7884673 | 0.0443832 |
| N-Acetylglucosamine 1-phosphate | 0.40345206 | 0.02683613 |
| 15-Hete | -1.3650093 | 0.00586567 |
| 4-Hydroxyphenylethanol | -1.0826613 | 0.00407554 |
| 2-C-Methyl-D-erythritol 2,4-cyclodiphosphate | 0.73286304 | 0.02359175 |
| Purine | 0.33640089 | 0.02080633 |

**Table S7. Differentially metabolites in serum samples between the ConA+M and ConA groups.**

| Name | log2FC | P value |
| --- | --- | --- |
| Allantoin | -0.5713228 | 0.04109449 |
| L-beta-Imidazolelactic acid | 0.85533142 | 0.03609807 |
| Lysopc 20:4 | -0.2353462 | 0.02324657 |
| Lysopc 18:3 | -0.1015656 | 0.03733134 |
| Glycerol 1-hexadecanoate | -0.3337966 | 0.02872141 |
| Dl-Threitol | -1.0710359 | 0.04510709 |
| Kynurenic acid | -1.9384894 | 0.01249639 |
| Asp-Phe methyl ester | -0.4839321 | 0.02220071 |
| 7-Methylguanine | -0.3474992 | 0.04138499 |
| 5beta-Androstan-17beta-ol-3-one | -0.6208417 | 0.0428678 |
| 5-Hydroxylysine | -0.862083 | 0.00438079 |
| 3-Guanidinopropanoate | -0.263705 | 0.03673697 |
| 3-amino isobutanoate | -0.8412506 | 0.01090312 |
| L-Kynurenine | -0.8652471 | 0.04688573 |
| 2,6-Dimethylaniline | -0.3549928 | 0.04670092 |
| 2-(Dimethylamino)Guanosine | -0.7629788 | 0.04109331 |
| 1-Oleoyl-Sn-Glycero-3-Phosphocholine | -0.266936 | 0.03888221 |
| 16alpha-Hydroxytestosterone | -0.342459 | 0.02332512 |
| 4-Methyl-2-Oxopentanoic Acid | 0.36749641 | 0.00491309 |
| Phe-Phe | -1.2516006 | 0.00055402 |
| 5'-Deoxy-5'-(methylthio)adenosine | -0.3092991 | 0.0182817 |
| Ethambutol | -0.4386161 | 0.03992591 |
| 2-Phenylethylamine | -1.9185187 | 0.02660751 |
| Diethylglutarate | -1.085186 | 0.0485057 |
| MGMG (18:2) | 0.48661441 | 0.00837732 |
| N-Carbamyl-L-glutamicacid | -0.9728745 | 0.04697312 |
| N-Acetyl-L-glutamic acid | -0.2978922 | 0.03338916 |
| Glu-Glu | -0.4189742 | 0.03344312 |
| 2-Oxo-3-methyl-butyrate | 0.76441628 | 0.03485587 |
| Delta-Tridecalactone | -0.7426545 | 0.036726 |
| L-allo-Isoleucine | -0.2602159 | 0.03504266 |
| Glycine | -0.4984753 | 0.0112491 |
| Phthalate | -0.3575917 | 0.04597996 |
| Tryptamine | -2.0697262 | 0.01395728 |
| Maleic Acid | -0.4432958 | 0.00944252 |
| trans-Cinnamic acid | -0.4351604 | 0.0312309 |
| 6-Methylmercaptopurine | -0.3568814 | 0.03624852 |
| Digitoxigenine | -1.0838564 | 0.02840011 |
| Indole-3-acetamide | -1.9575201 | 0.01962408 |
| 3-(2-Hydroxyphenyl)propionic acid | -0.3850851 | 0.0160058 |
| Cytarabine | 0.74568309 | 0.03008545 |
| Cuminaldehyde | -0.4433867 | 0.03437042 |
| Vidarabine | -1.02495 | 0.04692813 |
| L-Alanine | -0.4298368 | 0.02489023 |
| Pregnenolone | -0.8722592 | 0.03710326 |
| L-Arginine | 1.50046242 | 0.03352814 |
| Fumaric acid | -0.7907031 | 0.02241734 |
| L-Cysteine | 0.37411505 | 0.01124552 |
| 3-Hydroxyisovalerate | -0.6225684 | 0.04408106 |
| L-Malate | -0.767487 | 0.04069278 |
| N-acetyl-L-ornithine | -2.0580504 | 0.0170382 |
| Aminomalonic acid | -0.1198398 | 0.01378919 |
| D-Proline | -0.3972317 | 0.03104423 |
| 6-Phospho-D-gluconate | -0.522275 | 0.01953504 |
| Deoxyribose 5-Phosphate | -0.7105663 | 0.02999455 |
| L-Homocitrulline | -1.0408926 | 0.0008888 |
| N-Propionylglycine | -0.9678136 | 0.02225474 |
| L-Glutamate | -0.5434506 | 0.00121874 |
| hydroxyphenylpyruvate | -1.0319016 | 0.03711029 |
| Taurohyocholic acid | -1.2526137 | 0.00897844 |
| Tauro-alpha-Muricholic acid | -1.278813 | 0.00817973 |
| N-lactoyl-phenylalanine | -0.6611548 | 0.01383891 |
| Bisphenol A | -0.446989 | 0.04087485 |
| 2-Hydroxy-3-methylbutanoic acid | -0.9560612 | 0.02937455 |
| D-Phenylalanine | -0.2974836 | 0.04990199 |
| L-Glutamic acid | -0.6438171 | 0.01712759 |
| DL-Serine | -0.3462285 | 0.01906368 |
| 4-Hydroxyisoleucine | -0.6447213 | 0.01417437 |
| L-Thyroxine | -0.8037773 | 0.03292503 |
| Taurohyodeoxycholic acid | -1.4668011 | 0.01918602 |
| Taurodeoxycholic acid | -1.3806383 | 6.35E-05 |
| Tauroursodeoxycholic acid | -1.4676427 | 0.01977388 |
| Glycocholic acid | -1.2789666 | 0.01735308 |
| 3-Oxo-7alpha,12alpha-hydroxy-5beta-cholanoic acid | 2.53454653 | 0.01069056 |
| 23-Norcholic acid | -1.0983949 | 0.01113444 |
| Cytidine | 0.71572644 | 0.01311612 |
| Taurocholic acid | -1.0588006 | 0.04589035 |
| alpha-Ketoglutaric acid | -1.690438 | 0.00057514 |
| Inosine | 2.45734146 | 0.00122056 |
| Azelaic acid | -0.2343706 | 0.03307032 |
| 1-Methylhistidine | -0.3119786 | 0.04949713 |
| Sarcosine | -0.4300397 | 0.03722275 |
| Purine | -0.3301725 | 0.04948088 |
| Ne-(1-Carboxymethyl)-L-lysine | -0.4280205 | 0.0441596 |

**Table S8. Differentially metabolites in serum samples between the NR and CR groups.**

| Name | log2FC | P value |
| --- | --- | --- |
| Inosine | -3.208269 | 1.50E-05 |
| Hexanoylcarnitine | 0.61580683 | 0.00052159 |
| Allopurinol | -2.4401605 | 0.00081897 |
| L-Octanoylcarnitine | 0.70985506 | 0.00171762 |
| Decanoylcarnitine | 0.66662078 | 0.00575132 |
| 3-Methylsalicylate | -0.1689757 | 0.00594193 |
| Anthranilic acid | -0.997086 | 0.00657543 |
| Glutarylcarnitine | 0.40378367 | 0.01147619 |
| Dodecanoylcarnitine | 0.64848188 | 0.0122249 |
| Tetradecanoylcarnitine | 0.54408761 | 0.01735566 |
| S-Adenosyl-L-methionine | -2.2281446 | 0.0213354 |
| O-Acetyl-L-carnitine | 0.43350152 | 0.0232464 |
| Cytidine | 0.79957816 | 0.02400194 |
| Gly-Phe | 0.41148253 | 0.02452388 |
| N-Acetylalanine | 0.20180861 | 0.02487526 |
| Dimethyl fumarate | -0.344291 | 0.0253399 |
| SDMA | 0.2202176 | 0.02542139 |
| Acetylcarnitine | 0.41762845 | 0.02651902 |
| Hypoxanthine | -1.0224183 | 0.02680463 |
| 3-(3-Hydroxyphenyl)propanoic acid | 1.35840535 | 0.02879899 |
| Fosfomycin | 0.15638228 | 0.02894269 |
| L-Palmitoylcarnitine | 0.44041964 | 0.03053348 |
| Uric Acid | 0.28000506 | 0.03147283 |
| D-Glutamine | -0.181538 | 0.03261054 |
| Chenodeoxycholic acid | -0.8163159 | 0.03315547 |
| Isomaltose | -0.4188364 | 0.03657019 |
| 1,4-Dihydro-1-Methyl-4-Oxo-3-Pyridinecarboxamide | -0.8435044 | 0.04003508 |
| Oleoylcarnitine | 0.53532668 | 0.04150778 |
| Urocanic acid | 0.30633391 | 0.04246438 |
| Stearoylcarnitine | 0.39561338 | 0.04425111 |
| 4-Acetamidobutanoate | -0.2806551 | 0.04475133 |
| Anserine | -0.3802954 | 0.04570994 |
| Asp-glu | 0.33108777 | 0.04961795 |
| Sulfoacetic acid | 0.2849461 | 0.04992962 |

**Table S10. Clinical characteristics of patients with AIH included in the present study.**

| **Clinical characteristics** | **Amount (n=73)** |
| --- | --- |
| Age, years | 52.9 ± 12.5 |
| Female | 62/73 (84.9%) |
| Liver function indexes |  |
| TBil, μmol/L | 73.8 ± 73.5 |
| ALT, IU/L | 313.5 ± 347.0 |
| AST, IU/L | 333.2 ± 322.2 |
| ALB (g/L) | 37.3 ± 5.3 |
| GLB (g/L) | 40.3 ± 9.3 |
| Immunoglobulin |  |
| IgG, IU/L | 25.2 ± 9.0 |
| ANA (+, N%) | 67/73 (91.8%) |

TBil, total bilirubin; ALT, alanine aminotransferase; AST, aspartate aminotransferase; IgG, immunoglobulin G; ANA, antinuclear antibody.
